# Controller protein of restriction–modification system Kpn2I affects transcription of its gene by acting as a transcription elongation roadblock

**DOI:** 10.1093/nar/gky880

**Published:** 2018-10-08

**Authors:** Evgeny Klimuk, Ekaterina Bogdanova, Max Nagornykh, Andjela Rodic, Marko Djordjevic, Sofia Medvedeva, Olga Pavlova, Konstantin Severinov

**Affiliations:** 1 Center of Life Sciences, Skolkovo Institute of Science and Technology, Skolkovo, Russia; 2 Institute of Molecular Genetics, Russian Academy of Sciences, Moscow, Russia; 3 Waksman Institute of Microbiology, Piscataway, NJ 08854, USA; 4 Institute of Biochemistry and Physiology of Microorganisms, Russian Academy of Sciences, Puschino, Russia; 5 Faculty of Biology, University of Belgrade, Belgrade, Serbia; 6 Institute of Gene Biology, Russian Academy of Sciences, Moscow, Russia

## Abstract

C-proteins control restriction–modification (R–M) systems’ genes transcription to ensure sufficient levels of restriction endonuclease to allow protection from foreign DNA while avoiding its modification by excess methyltransferase. Here, we characterize transcription regulation in C-protein dependent R–M system Kpn2I. The Kpn2I restriction endonuclease gene is transcribed from a constitutive, weak promoter, which, atypically, is C-protein independent. Kpn2I C-protein (C.Kpn2I) binds upstream of the strong methyltransferase gene promoter and inhibits it, likely by preventing the interaction of the RNA polymerase sigma subunit with the -35 consensus element. Diminished transcription from the methyltransferase promoter increases transcription from overlapping divergent C-protein gene promoters. All known C-proteins affect transcription initiation from R–M genes promoters. Uniquely, the C.Kpn2I binding site is located within the coding region of its gene. C.Kpn2I acts as a roadblock stalling elongating RNA polymerase and decreasing production of full-length C.Kpn2I mRNA. Mathematical modeling shows that this unusual mode of regulation leads to the same dynamics of accumulation of R–M gene transcripts as observed in systems where C-proteins act at transcription initiation stage only. Bioinformatics analyses suggest that transcription regulation through binding of C.Kpn2I-like proteins within the coding regions of their genes may be widespread.

## INTRODUCTION

Type II restriction–modification (R–M) systems encode a restriction endonuclease that recognizes and cleaves specific DNA sequences and a methyltransferase that recognizes the same DNA sequence and methylates it first on one DNA strand - to produce hemimethylated DNA - and then on the other strand to produce fully methylated DNA (1). Methylation prevents site recognition by the endonuclease and thus protects DNA from cleavage. Bacterial cells carrying R–M system genes become resistant to infection by bacteriophages whose genomes contain unmethylated (unmodified) recognition sites. The genomes of many phages are devoid of recognition sites of R–M gene products commonly found in host bacteria (2), indicating that R–M systems have a profound influence on bacteriophages parasitizing on host bacteria. While this beneficial property doubtless contributed to wide dissemination of R–M systems in the eubacterial kingdom (3,4), multiple functions unrelated to phage defense may have also played a role (see (5) for review).

R–M systems genes (*res* and *met*) form tight clusters and are often carried on mobile genetic elements capable of horizontal spread between different bacterial species (6). Premature appearance of endonuclease activity upon entry of a genetic element carrying R–M system genes into a naïve host will lead to host DNA degradation (7,8). Therefore, active endonuclease should appear only after host DNA is completely methylated (9). Conversely, methyltransferase should appear early on to modify all available recognition sites as fast as possible. Excessive methyltransferase activity at later times can modify foreign DNA before it is cleaved, decreasing the level of protection. Thus, the initially robust synthesis of methyltransferase should decrease to lower steady-state levels when sufficient amounts of restriction endonuclease become available. Different strategies to accomplish this coupled genetic switch (delayed synthesis of restriction endonuclease in naïve host and decreased synthesis or methyltransferase at steady state) have been revealed. In some R–M systems transcription from weak *res* gene promoter is activated only after a certain threshold level of methyltransferase is reached (10–19). Incorporation of a recognition site into intrinsically strong *met* gene promoter consensus element allows sensing of the methylation state of DNA. Modification of this site leads to decreased *met* promoter activity (10,12,15,17,19). In other systems, methyltransferase binds to an operator site that overlaps with *met* promoter but is unrelated to the recognition site (13,14,16,18). The methyltransferase in such systems contains an additional DNA binding domain that recognizes the operator. In both cases, decreased transcription from *met* promoter leads to increased transcription from overlapping divergent *res* promoter.

A great number of Type II R–M systems encode an additional DNA binding C (controller) protein to ensure regulated *met* and *res* gene transcription (20–35). The C-protein gene typically precedes the *res* gene with which they form an operon (20,21,23,25,31–33). C-protein dimers bind to a C-box, a DNA sequence containing two C-protein dimer sites, located upstream of and partially overlapping with weak *c-res* operon promoter (36). For example, in the EcoRV system binding of C-protein dimer to a high-affinity distal site activates *c-res* operon transcription, leading to increased restriction endonuclease and C-protein synthesis (23). The high-affinity EcoRV C-protein binding site overlaps with strong *met* promoter, negatively regulating its activity and preventing excessive synthesis of methyltransferase (23). At high C-protein concentrations, an additional, low-affinity site becomes occupied, leading to inhibition of *c-res* operon transcription (23).

**Figure 1.**
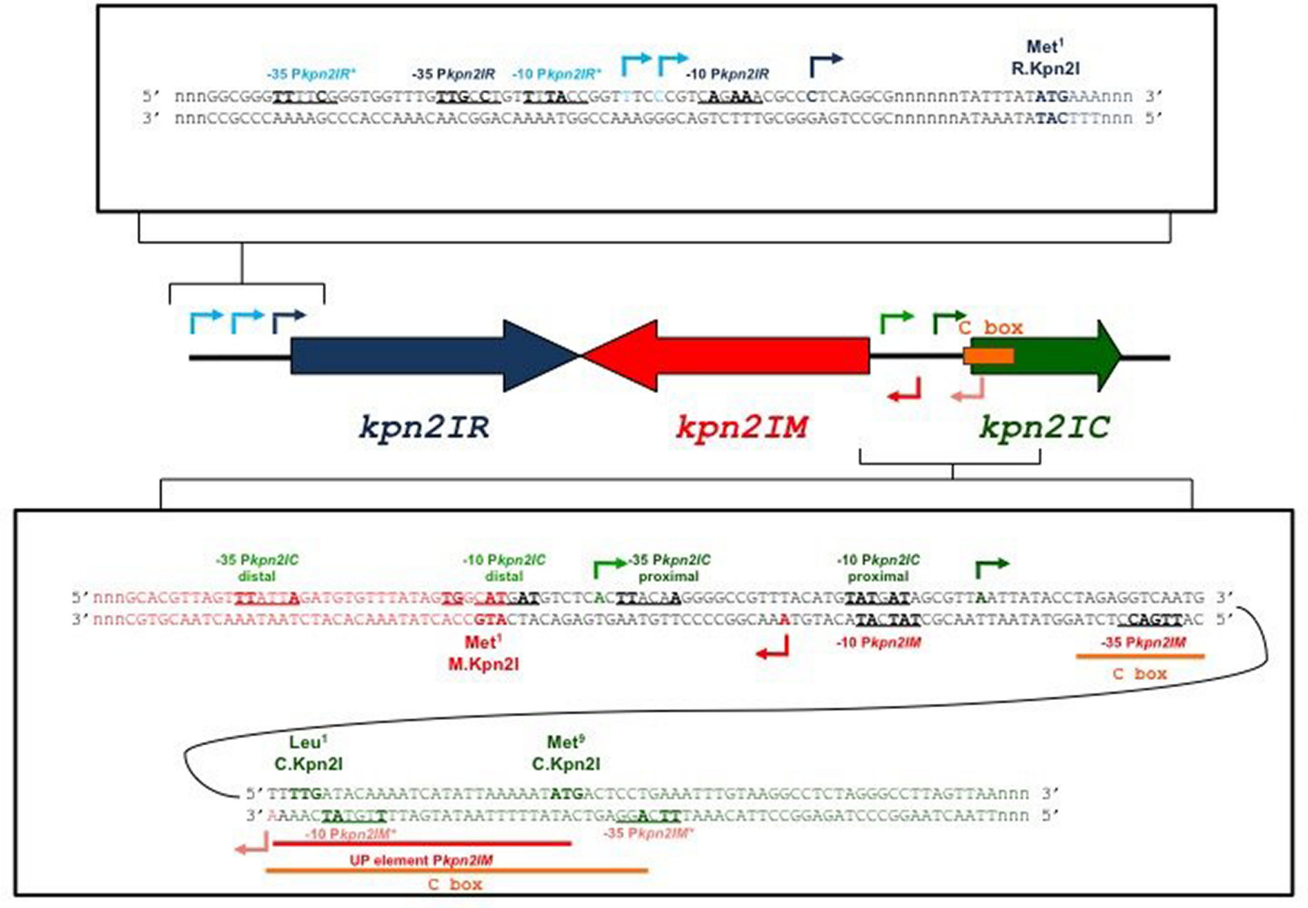
Genetic organization of restriction–modification system Kpn2I. In the middle of the figure, the Kpn2I genes are schematically shown by colored arrows, with arrow direction matching the direction of transcription. The DNA sequence upstream of the *kpn*2I*R* gene (both strands) is expanded at the top. The initiating codon of the *kpn*2I.*R* reading frame is indicated; the transcription start points of *kpn*2I*.R* promoters are shown by arrows, likely -10 and -35 promoter elements are underlined, nucleotides matching promoter element consensus are shown in bold. The sequence of the intergenic region between and the beginnings of oppositely transcribed *kpn*2I.*M* and *kpn*2I*.C* genes is expanded below. Initiating codons of both ORFs, transcription start points, and promoter consensus elements are indicated. Sequences downstream of initiating codons are colored to match the coloring scheme of the genes as shown in the middle of the figure (dark blue for *kpn*2I*.R*, red for *kpn*2I*.M*, dark green for *kpn*2I*.C*). Arrows indicating transcription start points are also colored to match the same coloring scheme. Arrows of darker shades indicate stronger promoters. The binding site of C.Kpn2I (as determined by DNase I and Exo III footprinting) is shown by an orange-colored horizontal line and marked ‘C box’). The likely UP element of *kpn*2I*.M* is marked.

In this work, we analyzed transcription regulation of C-protein-dependent R–M system Kpn2I. The genetic organization of this system has been reported previously (24). The three Kpn2I genes have an atypical arrangement (Figure 1). All three genes are transcribed separately; the *kpn*2I*.M* and *kpn*2I*.C* genes are transcribed divergently and are separated by a short intergenic region. The locations of Kpn2I genes promoters and C.Kpn2I binding sites have not been determined, and so it was not clear how the unusual architecture of this system allows coordinated expression of structural genes. In this work, we show that the *kpn*2I*.R* gene is constitutively expressed from a weak promoter, while *kpn*2I*.M* is expressed from a strong promoter whose activity is negatively regulated by C.Kpn2I. The *kpn*2I*.C* transcription initiates from divergent promoters that overlap with the *kpn*2I*.M* promoter. The negative feedback loop that prevents overproduction of C.Kpn2I is arranged in an unprecedented way: the C.Kpn2I binding site is located not upstream of *kpn*2I*.C* promoters but inside the *kpn*2I*.C* open reading frame; bound C.Kpn2I serves as a roadblock for transcription of a gene that encodes it. We incorporated experimental data in a mathematical model, which allowed predicting the dynamics of Kpn2I products synthesis. The model was used to *in silico* perturb key system regulatory features, in particular the unusual mode of control by C.Kpn2I, to assess its contribution to the system dynamics. We find that functionally, the dual transcription initiation-elongation control by C.Kpn2I leads to same consequences as the commonplace initiation-only control by C-proteins from other Type II R–M systems, showing that general functional constraints on Type II R–M systems components synthesis can be fulfilled by diverse molecular regulatory mechanisms and underscoring the versatility of C-proteins. Bioinformatics analyses suggest that transcription regulation through binding of C.Kpn2I-like proteins within the coding regions of their genes may be widespread.

## MATERIALS AND METHODS

### Bacteria strains, phages, and plasmids

*Escherichia coli* ER2267 (F′ *proA^+^B^+^ lacI^q^ Δ(lacZ)M15 zzf::mini-Tn*10 (Kan^R^)/*Δ(argF-lacZ)U169 glnV44* e14^−^(McrA^−^) *rfbD1 recA1 relA1 endA1 spoT1 thi-1 Δ(mcrC-mrr)114::IS10*) (New England Biolabs), *E. coli* XL1-Blue (F′*::Tn10 proA+B+ lacIq Δ(lacZ)M15/ recA1 endA1 gyrA96 (NalR) thi hsdR17 (rK– mK+) glnV44 relA1 lac*) (Stratagene) and *E. coli* HB101 (F^–^*Δ(gpt-proA)62leuB6glnV44 ara-14 galK2 lacY1 Δ(mcrC-mrr) rpsL20* (Str^R^) *xyl-5 mtl-1 recA13 thi-1*) (23) were used to study Kpn2I gene expression. *Escherichia coli* BL21(DE3) (*E. coli* B F^−^*dcm ompT hsdS(*r_B_^−^ m_B_^−^*) gal* λ(DE3)) (Stratagene) was used for recombinant proteins overproduction. *Escherichia coli* XL10-Gold (Δ (*mcrA)183 Δ(mcrCB-hsdSMR-mrr)173 endA1 supE44 thi-1 recA1 gyrA96 relA1 lac Hte* [F′ *proAB lacIq ZΔM15 Tn*10 (Tet^r^) Amy Cam^r^]) ultracompetent cells (Stratagene) were used for molecular cloning (37). All bacterial strains were grown in LB media (1% Bactotryptone, 1% NaCl, 0.5% yeast extract, with or without 1.5% Bactoagar) at 37°C with appropriate antibiotics. To test for activity of Kpn2I promoters cells were plated on McConkey agar base plates containing 1% galactose.

Plasmid pKpn2RM4.4 (24) carrying the entire Kpn2I system served as a template for PCR amplification of DNA fragments used for cloning, mutagenesis, and *in vitro* transcription. Plasmids pMetGalKpn, pCGalKpn and pResGalKpn are derivatives of the pFD51 plasmid (38) with the galactokinase gene (*galK*) placed under the control of *kpn*2I.*M, kpn*2I.*C* and *kpn*2I.*R* promoters, respectively. Plasmid pMetGalKpn contains a 250-bp PCR-amplified Kpn2I fragment (−128 to +122 with respect to the *kpn*2I.*M* promoter transcription start point); plasmid pCGalKpn contains a 213-bp PCR-amplified fragment (−164 to +49 with respect to the transcription start point of the proximal *kpn*2I*.C* promoter); pResGalKpn contains a 160-bp fragment (−70 to +90 with respect to the transcription start point of the *kpn*2I.*R* promoter).

Plasmid pCKpn177 was created by cloning a 512-bp PCR fragment containing the *kpn*2I*.C* gene under control of its own promoters between the ScaI and BamHI sites of plasmid pACYC177.

To generate pCkpn2I-6His plasmid for expression of hexahistidine-tagged C.Kpn2I, a 321-bp PCR fragment containing the *kpn*2I*.C* gene with incorporated flanking NdeI and EcoRI restriction sites was inserted between the NdeI and EcoRI sites of pET28 (Novagen).

To measure roadblocking effect *in vivo* plasmid ‘pET28_lux’ containing *luxCDABE* operon from *Photorhabdus luminescens* (39) under control of inducible *rhaB* promoter (Darya Esyunina, unpublished) and C.Kpn2I C-box positioned between luciferase operon and inducible promoter was created by ligation-independent cloning with Gibson Assembly Master Mix (New England Biolabs) according to the manufacturer's recommendations. The distance from transcription start site and C-protein binding site in this construct is the same as in the Kpn2I system.

Plasmid ‘pACYC_Ckpn’ was created by cloning a 102-bp PCR fragment containing T7A1 promoter (positions from –102 to +1 relative to the start of transcription) instead of the *kpn*2I*.C* gene promoters in plasmid pCKpn177 by ligation independent cloning with Gibson Assembly Master Mix (New England Biolabs) according to the manufacturer's recommendations.

Synthetic oligonucleotides (5′-CTAGAGGTCAATGTTTTGATACAAAATCATATTAAAAATATGAC-TCCTGAAATTTGTAAG-3′ and 5′-TCGACTTACAAATTTCAGGAGTCATATTTTTAATATGATTTTGT-ATCAAAACATTGACCT-3′ containing the Kpn2I C-box were annealed and ligated between the XbaI and SalI sites of pBend2 (40) to generate pBendCKpn2I. Sequences of other oligonucleotides used in this work are available from the authors upon request.

### Proteins

Hexahistidine-tagged C.Kpn2I (C.Kpn2I-6His) was overexpressed in *E. coli* BL21 (DE3) grown in LB medium containing 30 μg/ml kanamycin. Cells were grown at 37°C until OD600 reached 0.6 followed by induction with 1 mM isopropyl 1-thio-β-d-galactopyranoside and further growth for 2 h. Cells were harvested and frozen at −80°C. Cell pellets were resuspended in buffer A [20 mM Tris pH 8, 0.5 M NaCl] containing 1 mg/ml lysozyme and sonicated. The lysate was clarified by centrifugation at 16 000 × g for 1 h and filtration using a 0.45 μm filter. C.Kpn2I-6His was purified on a Chelating HP column (GE Healthcare) loaded with Ni^2+^ and equilibrated with buffer A. Wash cycles with buffer A containing 20 and 50 mM imidazole were performed before elution with 300 mM imidazole in buffer A. Protein was concentrated to 1 ml volume and applied to HiLoad 16/600 Superdex 75 pg column (GE Healthcare). Fractions with C.Kpn2I-6His were collected and concentrated to ∼10 mg/ml. Protein concentration was determined using the Bradford method with BSA as a standard. The purity of resulting protein is shown on a gel presented in Supplementary Figure S2.

C-terminally truncated σ^70^ variant σ^1–565^ was purified from cells transformed with the pCYB2_σ^1–565^ plasmid as described (41). The σ^70^ subunit and the RNAP σ^70^ holoenzyme were purified as described (42,43). Wild-type RNAP core enzyme and RNAP core enzyme containing C-terminally truncated subunit α^235^ were purified as described (44) (a generous gift of Dr Leonid Minakhin). GreA purified as described in reference (45) was a generous gift of Dr Daria Esyunina.

### Luciferase assay

*Escherichia coli* HB101 cells harbouring pET28_lux with or without compatible pACYC_Ckpn plasmid were grown in LB medium in the absence and in the presence of 0.1% rhamnose until OD600 = 0.8. At this time point, OD_600_ and luminescence values were recorded. Luminescence was measured with a Modulus Microplate Reader (Turner BioSystems, Inc.) with default parameters. Luminescence was normalized to OD_600_ values, experiments were independently repeated three times and the normalized luminescence values were averaged.

### RNA extraction, primer extension, and DNA sequencing

*Escherichia coli* ER2267 and HB101 cells harbouring Kpn2I promoter plasmids with or without compatible pCKpn177 plasmid were grown until OD600 = 0.4, cultures we rapidly chilled by adding ice, cells were collected by centrifugation. Cell pellets were either frozen in liquid nitrogen or immediately processed for RNA extraction using RNeasy Mini Kit (QIAGEN) according to the manufacturer's protocol. RNA samples were treated with DNase I (Fermentas). For primer extension reactions, 5 μg of total RNA was reverse-transcribed with 100 U of SuperScript III enzyme from First-Strand Synthesis Kit for RT-PCR (Invitrogen) according to the manufacturer's recommendations in the presence of 1 pmol of 5′ [^32^P] end-labelled specific primer (5′-CTCTGCTGCCTGTTCTGCGG-3′). Reaction products were treated with RNase H, precipitated with ethanol, dissolved in 7M urea-formamide loading buffer and resolved on 7% polyacrylamide 7 M urea sequencing gels. The products of Sanger sequencing reactions performed with the same end-labelled primers and appropriate plasmids as templates using *fmol* DNA Cycle Sequencing System (Promega) were run alongside primer extension reactions as markers. Reaction products were revealed using PhosphoImager (Molecular Dynamics).

### Electrophoretic mobility shift assays with C-box fragments

The reactions contained, in 10 μl of reaction buffer [40 mM Tris–HCl (pH 8.0), 40 mM KCl, 10 mM MgCl_2_], 0, 15, 30, 60, 120, 250, 500 or 1000 nM of C.Kpn2I-6His and 100 nM of 42-bp (full C-box) or 23/25-bp (halves of C-box) DNA fragments obtained by annealing of appropriate complementary oligonucleotides. Reactions were incubated for 10 min at 37°C, combined with 2 μl of loading buffer (50% glycerol, 0.05% bromophenol blue) and immediately loaded onto 15% native polyacrylamide gels. After electrophoresis at 200 V for 1 h at room temperature, reaction products were stained with Ethidium Bromide (MP Biomedicals) and visualized using Gel Doc EZ Imager (Bio-Rad).

### DNA-bending assay

∼150-bp DNA fragments containing the Kpn2I C-box were isolated and radioactively end-labeled with [γ-^32^P]ATP and T4 polynucleotide kinase after digestion of the pBend_CKpn2I plasmid with various restriction enzymes. Binding of C.Kpn2I-6His to various fragments and separation by native PAGE was performed as described above. Briefly, the reactions contained, in 10 μl of reaction buffer [40 mM Tris–HCl (pH 8.0), 40 mM KCl, 10 mM MgCl_2_], 40 nM of radioactively labeled DNA fragments and where needed 200 nM of C.Kpn2I-6His. Reactions were incubated for 10 min at 37°C, combined with 2 μl of loading buffer (50% glycerol, 0.05% bromophenol blue) and immediately loaded onto 8% native polyacrylamide gels. Products were visualized by autoradiography.

### Footprinting and *in vitro* transcription reactions

The reactions contained, in 10 μl of reaction buffer, 40 nM of 200-bp *kpn*2I DNA fragment (−140 to +60 with respect to the transcription start point of the proximal *kpn*2I*.C* promoter end-labeled with [γ-^32^P]ATP and T4 polynucleotide kinase) and where needed 200 nM of C.Kpn2I-6His. After a 10-min incubation at 37°C, 0.05 U DNase I (Worthington) was added and incubation was continued for another 45 s. Reactions were stopped by the addition of 20 μl stop buffer (1% SDS, 200 mM EDTA, pH 8.0, 50 μg/ml calf thymus DNA) and ammonium acetate to the final concentration of 1 M. Samples were precipitated with ethanol, dried and resuspended in 8 μl of 7 M urea–formamide loading buffer. G+A sequencing reactions were performed with the same (unlabelled) DNA fragments and appropriate end-labeled primers using *fmol* DNA Cycle Sequencing System (Promega) for mixture of G and A nucleotides and were run alongside primer extension reactions as markers. Samples were applied on 6% polyacrylamide 7 M urea sequencing gels and products were revealed using PhosphorImager.

Transcription reactions were set in 10 μl and 40 nM of transcription templates with *kpn*2I promoters, *gal*P1 (44), or T7 A1 (46) and 100 nM *E. coli* RNAP σ^70^ holoenzyme (or its mutant versions) in a buffer containing 40 mM Tris–HCl, pH 8.0, 40 mM KCl, 10 mM MgCl_2_, 1 mM DTT, 50 μg BSA, 5% glycerol, 10 U RiboLock RNase Inhibitor (Thermo Scientific). 200 nM C.Kpn2I-6His or 1 μM GreA were added to reactions when appropriate. After 5–10 min incubation at 37°С, the reactions were supplemented with 2 μl of nucleotide hot mix (2 mM АТP, GТР, and СТР, 500 μМ UТP and 0.5 μCi [α-^32^P]-UТP (3000 Ci/mmol) and incubated for additional 10 min at the same temperature. Reactions were terminated by the addition of 10 μl of formamide-containing loading buffer and loaded on a 7% polyacrylamide 7 M urea sequencing gels.

KMnO_4_ probing was conducted under conditions used in *in vitro* transcription assays with kpn2I DNA fragment (−140 to +60 with respect to the transcription start point of the proximal *kpn*2I*.C* promoter) as a template. Complexes were treated with 1 mM KMnO_4_ for 30 s at 37°C followed by the addition of β-mercaptoethanol and ammonium acetate to the final concentrations of 1 M and 0.3 M, respectively. Samples were precipitated by ethanol, dried and resuspended in 90 μl H_2_O. After the addition of 10 μl piperidine, samples were incubated at 90°C for 20 min, precipitated with ethanol, dried and resuspended in 8 μl of 7 M urea–formamide loading buffer.

ExoIII footprinting was conducted at same conditions as described (47). Samples were applied to 6% polyacrylamide 7 M urea sequencing gel and revealed by PhosphorImager (Molecular Dynamics).

### Bioinformatics methods

C.Kpn2I-related proteins were identified using BLASTP against nr70 (e-value < 10e^−5^). For each of 285 found unique homologs, nucleotide sequences extending 50 bp upstream to 50 bp inside the annotated start of the gene were downloaded and conserved motifs were predicted by MEME (48). Seventy one of retrieved sequences contained the C.Kpn2I motif. For phylogenetic tree construction, a subset of eight XRE-domain proteins (49) whose genes contained predicted C.Kpn2I binding sites were used. This subset was combined with sequences of C-proteins (from Ref. (49)) and multiple alignment was created with PROMALS3D using default parameter setting (51). The alignment was cut by trimAl using ‘gappyout’ option (52). The maximum likelihood tree was built with PhyML (53). The tree was collapsed by support value 0.7 and visualized in iTOL (54).

### Modeling transcription regulation of Kpn2I genes

#### General model

Transcription regulation model that takes into account possible configurations of the (common) *kpn*2I*.M* and *kpn*2I*.C* regulatory region is described by the following binding reactions, and the associated equilibrium dissociation constants $(K):$

(2.1) \begin{eqnarray*} &&[{\rm RNAP}] + [{\rm DNA}]\,\,\,\raise2pt\hbox{$\displaystyle\mathop{-\!\!\!-\!\!\!-\!\!\!\longrightarrow}$}\raise-2pt\hbox{$\displaystyle\mathop{\longleftarrow\!\!\!-\!\!\!-\!\!\!-}_{K_{P.M}}$}\,\,\,[{\rm RNAP}\sim{\rm P.M}],\nonumber \\ &&[{\rm RNAP}] + [{\rm DNA}]\,\,\,\raise2pt\hbox{$\displaystyle\mathop{-\!\!\!-\!\!\!-\!\!\!\longrightarrow}$}\raise-2pt\hbox{$\displaystyle\mathop{\longleftarrow\!\!\!-\!\!\!-\!\!\!-}_{K_{P.C}}$}\,\,\,[{\rm RNAP}\sim{\rm P.C}],\nonumber \\ &&{p_C} + {p_C}\,\,\,\raise2pt\hbox{$\displaystyle\mathop{-\!\!\!-\!\!\!-\!\!\!\longrightarrow}$}\raise-2pt\hbox{$\displaystyle\mathop{\longleftarrow\!\!\!-\!\!\!-\!\!\!-}_{K_{D}}$}\,\,\,[{\rm D}],\nonumber \\ &&[{\rm D}] + [{\rm DNA}]\,\,\,\raise2pt\hbox{$\displaystyle\mathop{-\!\!\!-\!\!\!-\!\!\!\longrightarrow}$}\raise-2pt\hbox{$\displaystyle\mathop{\longleftarrow\!\!\!-\!\!\!-\!\!\!-}_{K_{R}}$}\,\,\,[{\rm D}\sim{\rm R}],\nonumber \\ &&[{\rm D}\sim {\rm R} ] + [{\rm D}]\,\,\,\raise2pt\hbox{$\displaystyle\mathop{-\!\!\!-\!\!\!-\!\!\!\longrightarrow}$}\raise-2pt\hbox{$\displaystyle\mathop{\longleftarrow\!\!\!-\!\!\!-\!\!\!-}_{K_{L}}$}\,\,\,[{\rm T}\sim{\rm LR}],\nonumber \\ &&[{\rm T}\sim {\rm LR} ] + [{\rm RNAP}]\,\,\,\raise2pt\hbox{$\displaystyle\mathop{-\!\!\!-\!\!\!-\!\!\!\longrightarrow}$}\raise-2pt\hbox{$\displaystyle\mathop{\longleftarrow\!\!\!-\!\!\!-\!\!\!-}_{K_{P.C}}$}\,\,\,[{\rm RNAP}\sim{\rm P.C/T}\sim{\rm LR}],\nonumber\\ \end{eqnarray*}

where concentrations of reactants and reaction products are denoted as follows: [RNAP]-RNA polymerase; [DNA]-DNA containing the regulatory region; [RNAP∼P.M] - RNAP bound to the *kpn*2I*.M* promoter; [RNAP∼P.C]-RNAP bound to the *kpn*2I*.C* promoter(s); ${p_C}$-C.Kpn2I monomers; [D]-C.Kpn2I dimers; [D∼R]-C.Kpn2I dimer bound to the right high-affinity binding site; [T∼LR]-C.Kpn2I tetramer (two dimers) bound to both binding sites of the C-box; [RNAP∼P.C/T∼LR]-RNAP bound to the *kpn*2I*.C* promoter in the presence of a bound C.Kpn2I tetramer. To derive the configuration statistical weights introduced in the Results section, RNAP concentration and appropriate equilibrium dissociation constants were absorbed into few parameters $(f,g\, {\rm{and}}\, h):$

(2.2) \begin{eqnarray*} && f = [{\rm RNAP \sim P.M}]/[{\rm DNA}] = [{\rm RNAP]/}K_{P.M} , \nonumber\\ && g = [{\rm RNAP \sim P}{\rm .C}]/[{\rm DNA]} = [{\rm RNAP}]/K_{P.C} ,\nonumber\\ && p_C^4 /h = [{\rm T \sim LR}]/[{\rm DNA]} = p_C^4 /(K_D^2 \cdot K_L \cdot K_R ). \end{eqnarray*}

While *kpn*2I*.M* promoter transcription activity is determined only by the rate of transcription initiation (Equation (3.2)), *kpn*2I*.C* promoter transcription activity ${\tilde{\varphi }_C}({\tilde{p}_C})$ (Equation (3.3)), as stated in Results, is proportional to the product of the probability that a transcript is initiated:

(2.3) \begin{equation*}\left( {\frac{{\tilde{\alpha } \cdot g \cdot \left( {1 + \tilde{p}_C^4(\tau )} \right)}}{{1 + f + g + \left( {1 + g} \right) \cdot \tilde{p}_C^4(\tau )}}} \right)\end{equation*}

and the probability that the transcript is completely elongated:

(2.4) \begin{eqnarray*}\left( {\frac{{1 + f + g}}{{1 + f + g + \left( {1 + g} \right) \cdot \tilde{p}_C^4(\tau )}} + \beta \frac{{\left( {1 + g} \right) \cdot \tilde{p}_C^4(\tau )}}{{1 + f + g + \left( {1 + g} \right) \cdot \tilde{p}_C^4(\tau )}}} \right){\rm{ }}.\nonumber \\ \end{eqnarray*}

Upon initiation, the transcript is completely elongated if C.Kpn2I tetramer is not bound to DNA (the left term in (2.4)), or if C.Kpn2I is bound but RNAP reads through the roadblock (the right term in (2.4))—note that $\beta$ is the probability of read-through, while the term multiplying $\beta$ is the probability of C.Kpn2I being bound to DNA.

#### Rescaling the model

Inferring the main features of Kpn2I system establishment dynamics does not require handling the actual molecule numbers. We therefore rescaled the model quantities to reduce the number of parameters in the model. Specifically, time is multiplied by the transcript degradation rate $({\lambda _t}),$ to obtain non-dimensional $\tau$, while $\tilde{k}$ and $x$ were obtained by dividing, respectively, the rate of translation and the rate of protein degradation with ${\lambda _t}.$ The absolute amounts of transcripts $({m_i})$ and proteins $({p_i})$ are rescaled as follows: ${\tilde{m}_i} = {{{m_i}} \mathord{/ {\vphantom {{{m_i}} {\sqrt[4]{h}}}} } {\sqrt[4]{h}}},$${\tilde{p}_i} = {{{p_i}} \mathord{/ {\vphantom {{{p_i}} {\sqrt[4]{h}}}} } {\sqrt[4]{h}}},$ while the rate at which RNAP leaves the promoter during transcription $(\alpha )$ is rescaled to obtain $\tilde{\alpha } = {\alpha \mathord{/ {\vphantom {\alpha {( {{\lambda _t} \cdot \sqrt[4]{h}} )}}} } {( {{\lambda _t} \cdot \sqrt[4]{h}} )}}.$

#### Estimating parameter values

**Figure 2.**
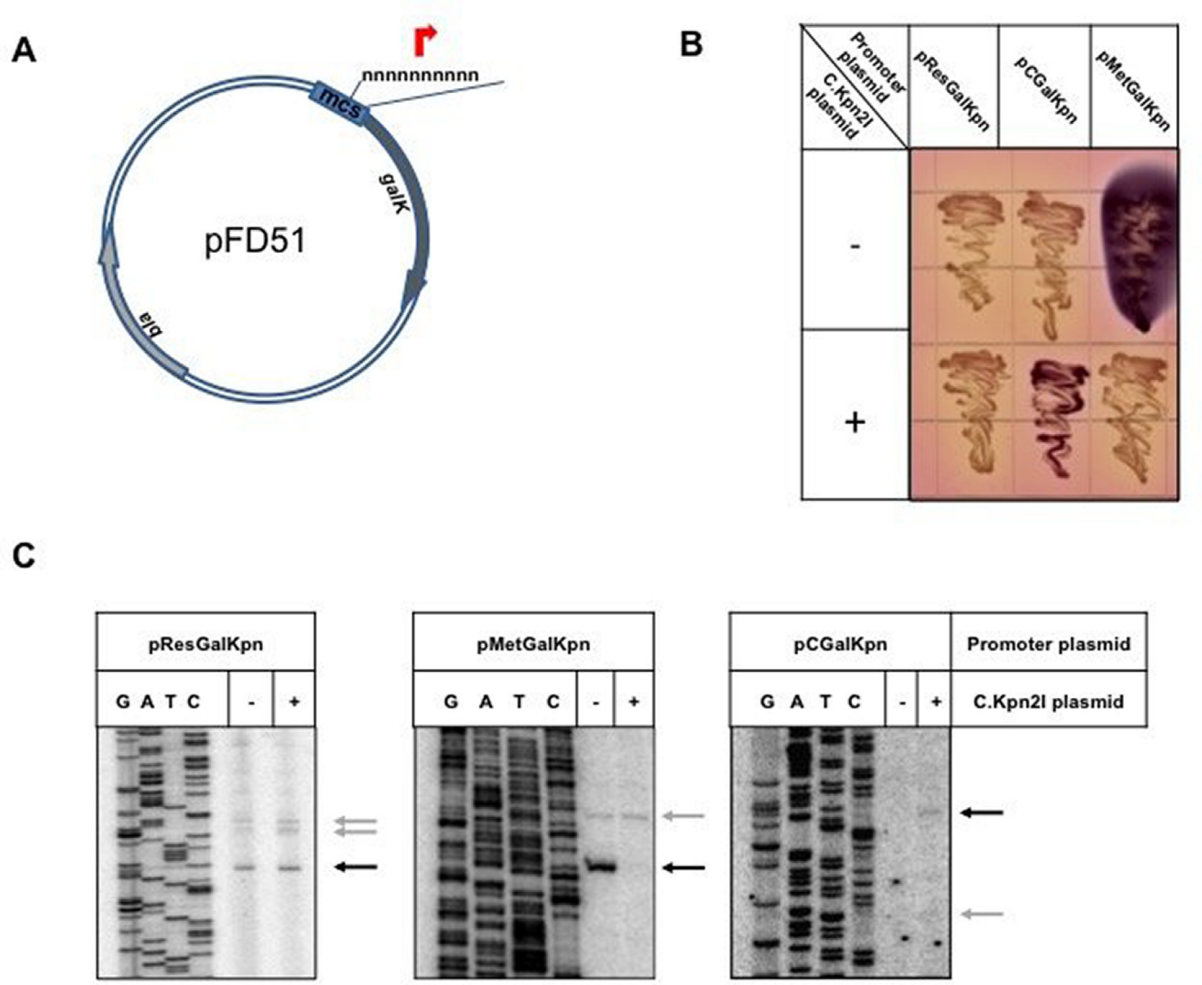
*In vivo* mapping of Kpn2I promoters. (**A**) The pFD51 promoter-trap plasmid with its multiple cloning site (mcs) used to clone DNA fragments in front of promoterless *galK* gene is schematically shown. (**B**) Overnight growth of *E. coli* cells harboring pFD51-based plasmids with Kpn2I promoters cloned upstream of *galK* on a McConkey agar plate. Cells in some cultures also contained a compatible plasmid producing C.Kpn2I. Colonies formed by cells carrying empty vector pFD51 are of the same (white) color as colonies of cells carrying pCGalKpn (not shown). (**C**) RNA was purified from *E. coli* cell cultures whose growth is shown in B and subjected to primer extension reaction with a *galK*-specific primer. Primer extension end points are marked with horizontal arrows and correspond to transcription start points shown in Figure 1.

We used the following data in estimating the model parameters: (i) Figure 2C, i.e., intensities of gel bands that correspond to transcript amounts initiated from a given promoter in the absence (‘−’ lanes) and in the presence (‘+’ lanes) of C.Kpn2I. (ii) Figure 6A, (provides a measure of the roadblock, i.e. the effect of C.Kpn2I on the amount of full-length *kpn*2I*.C* transcripts synthesis, which in turn allows extracting the $\beta$ parameter, see below. (iii) An assumption that *steady-state* amounts of *kpn*2I.*M, kpn*2I.*C* and *kpn*2I.*C* transcripts are approximately equal, which allows us to more directly compare their dynamics and is supported by observed intensities of primer extension products (see Figure 5A).

Using the data described under (i) above, i.e. the ratio of transcript amounts (measured by the band intensities) in the absence and in the presence of C.Kpn2I, which corresponds to the ratio of the transcription initiation rates $({\varphi _i})$, for, respectively, *kpn*2I*.M* and *kpn*2I*.C* promoters:

(2.5) \begin{equation*}\begin{array}{@{}l@{}} \frac{{\tilde{\alpha } \cdot f}}{{1 + f + g}} \cdot {\left( {\frac{{\tilde{\alpha } \cdot f}}{{1 + f + g + \left( {1 + g} \right) \cdot \tilde{p}_{Ceq}^4}}} \right)^{ - 1}} = 86.229{\rm{ ,}}\\ \frac{{\tilde{\alpha } \cdot g}}{{1 + f + g}} \cdot {\left( {\frac{{\tilde{\alpha } \cdot g \cdot \left( {1 + \tilde{p}_{Ceq}^4} \right)}}{{1 + f + g + \left( {1 + g} \right) \cdot \tilde{p}_{Ceq}^4}}} \right)^{ - 1}} = 0.263{\rm{ }}. \end{array}\end{equation*}

Furthermore, we use the data described under (ii) above, i.e., the ratio of band intensities corresponding to the ratio of the transcription activities with and without the roadblock (i.e. to the probability provided by (2.4)):

(2.6) \begin{equation*}\frac{{1 + f + g + \beta \cdot \left( {1 + g} \right) \cdot \tilde{p}_{Ceq}^4}}{{1 + f + g + \left( {1 + g} \right) \cdot \tilde{p}_{Ceq}^4}} = 0.44{\rm{ }}.\end{equation*}

From the condition introduced under (iii), i.e. that at equilibrium ${\tilde{\varphi }_M}({\tilde{p}_{Ceq}}) \approx {\tilde{\varphi }_C}({\tilde{p}_{Ceq}}) \approx {\tilde{\varphi }_{Req}}$ we obtain:

(2.7) \begin{equation*}\frac{f}{g} = \frac{{\left( {1 + \tilde{p}_{Ceq}^4} \right) \cdot \left( {{f \mathord{\left/ {\vphantom {f {\left( {1 + g} \right)}}} \right. } {\left( {1 + g} \right)}} + 1 + \beta \cdot \tilde{p}_{Ceq}^4} \right)}}{{{f \mathord{\left/ {\vphantom {f {\left( {1 + g} \right) + 1}}} \right. } {\left( {1 + g} \right) + 1}} + \tilde{p}_{Ceq}^4}}{\rm{ }}.\end{equation*}

We also take into account the standard equilibrium condition:

(2.8) \begin{eqnarray*}{\tilde{\varphi }_C}({\tilde{p}_{Ceq}}) &&= \frac{x}{{\tilde{k}}} \cdot \tilde{p}_{Ceq}^{} \nonumber\\ &&= \frac{{\tilde{\alpha } \cdot g \cdot \left( {1 + \tilde{p}_{Ceq}^4} \right) \cdot \left( {1 + f + g + \beta \cdot \left( {1 + g} \right) \cdot \tilde{p}_{Ceq}^4} \right)}}{{{{\left( {1 + f + g + \left( {1 + g} \right) \cdot \tilde{p}_{Ceq}^4} \right)}^2}}}{\rm{ ,}}\end{eqnarray*}

Together, all the conditions stated above (Equations (2.5)–(2.8)) allow determining an unambiguous combination of model parameters: $f = 2.9,$$g = 0.02,$$\tilde{\alpha } = 28,$$\beta = 0.44.$

Finally, $\tilde{k}$ and $x$ were set to standard literature values (55): $\tilde{k} = 3$ (so that a transcript is translated 3 times during its lifetime) and $x = 1/6$ (so that proteins are degraded 6 times slower than transcripts).

## RESULTS

### Mapping of Kpn2I promoters

The pKpn2I plasmid contains the entire Kpn2I R–M system cloned on a low-copy number (15–20 copies per cell) pACYC vector (24). pKpn2I is stably maintained in *E. coli* cells and provides resistance to phage infections (24), indicating that sufficient amounts of restriction endonuclease are produced. The λvir phage from rare plaques that formed on lawns of cells harboring pKpn2I was no longer restricted by these cells, and was therefore modified. To map Kpn2I promoters total RNA prepared from cells harboring pKpn2I was used in primer extension experiments with Kpn2I gene-specific primers. However, in no case primer extension products were observed, suggesting that the basal level of expression is below the limit of detection of the method used. For subsequent experiments, we therefore subcloned fragments expected to contain Kpn2I genes promoters upstream of promoterless *galK* gene of pBR322-based medium-copy number (50-100 copies per cell) pFD51 plasmid ((38), Figure 2A).

#### The *kpn*2I*.R* promoters

Cells harboring the pResGalKpn plasmid containing a DNA fragment upstream of *kpn*2I*.R* cloned in front of promoterless *galK* formed white-color colonies on McConkey agar plates (Figure 2B). Primer extension analysis with a *galK-*specific primer revealed one major and two closely spaced minor primer extension products (Figure 2C). Inspection of Kpn2I sequence upstream of major primer extension end point revealed a partial (three out of six) match with the –10 promoter element consensus sequence TATAAT (56) and a four out of six match with the –35 promoter element consensus sequence TTGACA (56) (Figure 1). The partially matching sequences were separated by 18 bp (optimal distance 17–18 bp, (56)). Putative promoter elements for transcripts corresponding to closely positioned minor primer extension products were also identified and had, as expected, poorer matches to consensus sequences (two out of six for both –10 and –35 elements, Figure 1).

Colonies formed by cells carrying pResGalKpn and compatible *kpn*2I.*C* expression plasmid remained white on McConkey agar and primer extension analysis indicated that transcription initiation from *kpn*2I*.R* promoters was unaffected by *kpn*2I.*C* expression (Figure 2B). We conclude, in agreement with earlier data (24), that *kpn*2I*.R* promoters are weak and C.Kpn2I-independent.

#### The *kpn*2I*.M* promoters

A pFD51-based plasmid pMetGalKpn carrying a Kpn2I fragment located between *kpn*2I*.M* and *kpn*2I*.C* cloned so that the direction of transcription of the *galK* gene matched that of *kpn*2I*.M* gene transcription direction was created. Cells carrying pMetGalKpn formed colonies of deep purple color on McConkey plates (Figure 2B). When the *kpn*2I.*C* expression plasmid was introduced in these cells, white color colonies were formed (Figure 2B). Primer extension analysis of RNA prepared from cells carrying pMetGalKpn, with or without *kpn*2I*.C* expression plasmid, was performed using *galK*-specific primers. A major primer extension product was revealed in cells lacking the *kpn*2I*.C* expression plasmid (Figure 2C). Upstream of the primer extension product 5′ end there is a sequence with good matches (five out of six) to the –10 and –35 promoter consensus elements separated by 18 bp of intervening DNA. These elements thus constitute the major *kpn*2I*.M* promoter. No primer extension band corresponding to this promoter was observed in the presence of *kpn*2I*.C* expression plasmid, indicating that it is repressed by C.Kpn2I.

In addition to major primer extension product, a minor primer extension band located slightly upstream was observed. The intensity of this band was not dependent on the presence of *kpn*2I*.C*. Upstream of the primer extension product 5′ end there is a sequence with three out of six matches to the –10 and –35 promoter consensus elements separated by 19 bp of intervening DNA.

#### The *kpn*2I*.C* promoters

A pFD51-based plasmid pCGalKpn carrying a Kpn2I fragment located between *kpn*2I.M and *kpn*2I.C was created so that the direction of transcription of *galK* matched that of *kpn*2I.C gene transcription. Cells carrying pCGalKpn formed white colonies on McConkey plates (Figure 2B). In the presence of *kpn*2I*.C* expression plasmid the color of colonies was purple (Figure 2B). Primer extension analysis of RNA prepared from both kinds of cells with *galK*-specific primers revealed two faint primer extension products only in the presence of the *kpn*2I*.C* plasmid (Figure 2C). The 5′ end of RNA corresponding to the more prominent higher mobility band is located 20 bp downstream of the start point of divergent major *kpn*2I*.M* promoter. Upstream of the primer extension product 5′ end there is a sequence with four out of six matches to the –10 and three out of six matches to the –35 promoter consensus elements. The –10 element is also preceded by a TG element found in extended –10 promoters. We call this promoter *kpn*2I*.C_dist*. The weaker primer extension product has a 5′ end located 16 bp upstream of the *kpn*2I*.M* promoter start point. We call this promoter *kpn*2I*.C_prox*. It also contains recognizable –10 and –35 promoter elements. However, the -10 element of *kpn*2I*.C_prox* overlaps with the -10 element of the much stronger *kpn*2I*.M* promoter, which is the likely cause of its negligible activity.

### Mapping the C.Kpn2I DNA binding site

**Figure 3.**
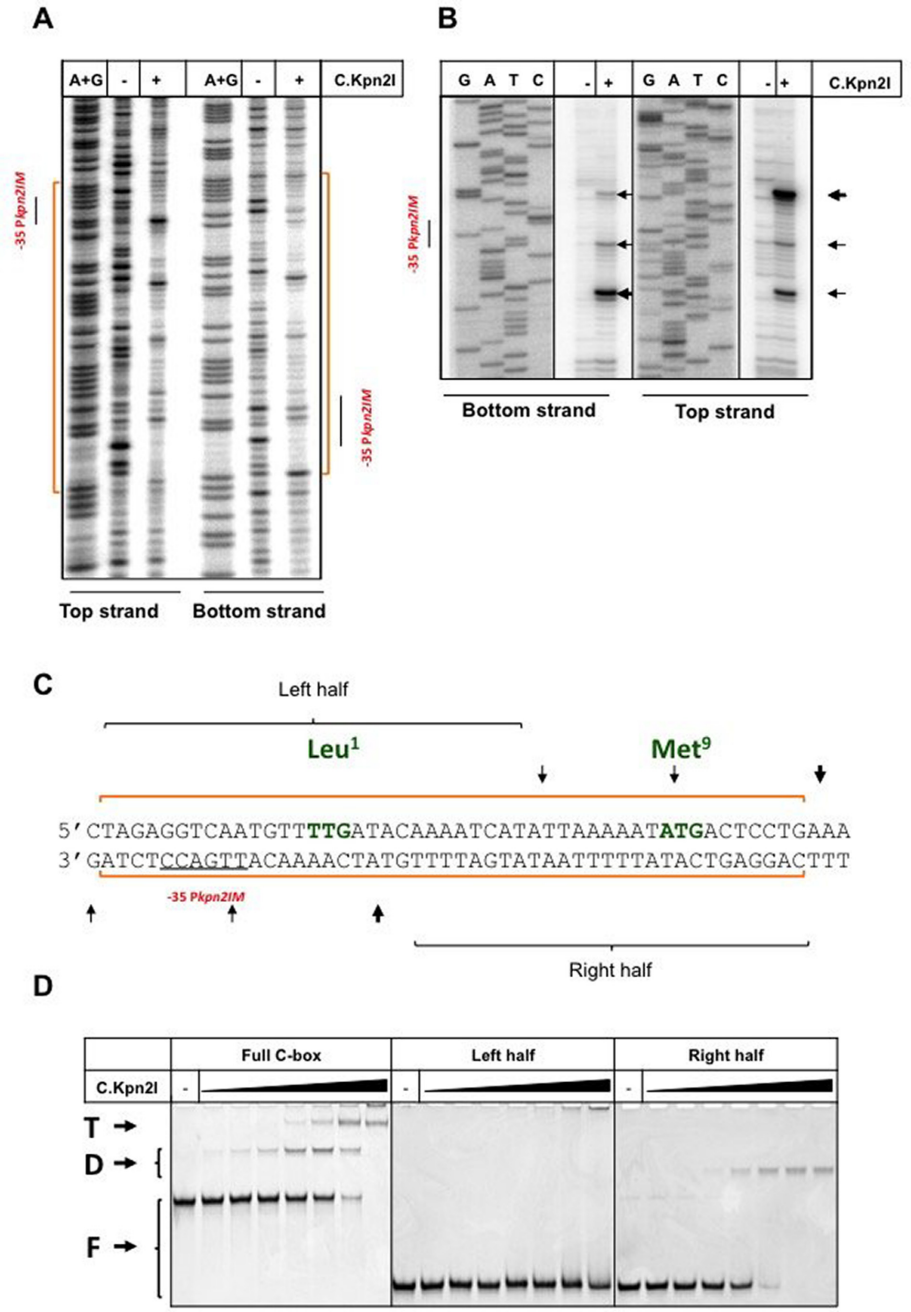
Mapping of the C.Kpn2I binding site. DNase I (**A**) and Exo III (**B**) footprinting of C.Kpn2I complexes formed on a DNA fragment separating *kpn*2I*.M* and *kpn*2I*.C*. Results obtained with DNA fragment labeled at either top or bottom strands (see Figure 1) in the presence or in the absence of C.Kpn2I are shown. Areas protected by C.Kpn2I from DNase I digestion are indicated by blue-colored brackets at both sides of the gel shown in panel A (also shown by orange line at the bottom of Figure 1). The positions of Exo III stalling points during DNA digestion in B are shown by horizontal arrows. (**C**) Summary of footprinting results. The positions of Exo III stalls and areas of DNA protection on both strands are shown by vertical arrows and horizontal blue lines, respectively. The –35 element of the *kpn*2I*.M* promoter is underlined. The initiating TTG (Leu^1^) codon of *kpn*2I*.C* and the Met^9^ ATG are indicated (see text for details). The left- and right half sites fragments used in EMSA experiments are indicated. (**D**) A double-stranded radioactively-labeled Kpn2I DNA fragment shown in C (‘full C-box’) or shorter fragments corresponding to its left- and right-hand side halves were combined with increasing amounts of C.Kpn2I and reaction products were resolved by native PAGE. ‘F’ indicates free DNA, ‘D’—a complex likely bound to C.Kpn2I dimer, ‘T’—a complex bound to C.Kpn2I tetramer.

Previous work showed that C.Kpn2I negatively regulates *kpn2I.M* expression but has no effect on *kpn2I.R* expression (24). The effect of C.Kpn2I on its own gene expression was not studied and its DNA binding site(s) was not defined. To map the C.Kpn2I binding site, increasing amounts of C.Kpn2I were combined with DNA fragment containing the intergenic region separating *kpn*2I*.M* and *kpn*2I*.C* or a fragment containing the sequence upstream of the *kpn*2I*.R* gene and complexes were examined by electrophoretic mobility shift assay (EMSA). The results revealed no binding to *kpn*2I*.R* fragment and robust binding to *kpn*2I*.M-kpn*2I*.C* intergenic region. To map the C.Kpn2I binding site we used DNase I and ExoIII footprinting in the presence of high concentrations of C.Kpn2I. C.Kpn2I protected both strands of DNA from position –14 to +34 with respect to the annotated *kpn*2I*.C* translation start point from DNAse I digestion (Figure 3A and C). Within the protected region, several regularly interspaced bands hypersensitive to DNAse I were observed. The ExoIII footprint revealed that the binding of C.Kpn2I causes major stops at positions +5 and +35 with respect to *kpn*2I*.C* translation start point as well as several minor stops further inside the binding site (Figure 3B and C).

A short DNA fragment encompassing the area protected by C.Kpn2I from DNase I digestion was tested in EMSA experiment with increasing concentrations of C.Kpn2I (Figure 3D, left). Two types of complexes were observed. By analogy with other C-proteins (57,58), we infer that the higher mobility complex corresponds to a single C.Kpn2I dimer bound to DNA, while the lower mobility complex corresponds to a tetramer/two dimers bound. The site of DNA to which C.Kpn2I binds lacks obvious paired inverted repeats present in other known C-protein binding sites (27). Yet the EMSA results suggest that there must be two binding sites, one with higher affinity than the other. To map individual sites more precisely, EMSA experiments with double-stranded oligonucleotides corresponding to halves of the full site were performed (Figure 3D). The right-hand side fragment bound C.Kpn2I well, forming a single complex, presumably corresponding to one dimer bound. There was no binding detected to the left-hand side fragment.

The results of C.Kpn2I binding site mapping is highly surprising, since most of protected area is located within the *kpn*2I*.C* ORF, downstream of the annotated start codon (Figure 3C). This is an unprecedented situation, since all predicted or known C-protein binding sites are located upstream of C-protein genes promoters (27,59). The annotated start codon of *kpn*2I*.C* is TTG (24). An in-frame ATG codon is located seven codons downstream (Figures 1 and 3C). This ATG codon is located at the outer edge of the area protected by C.Kpn2I and could conceivably be used as an initiation codon for C.Kpn2I polypeptide. To test this possibility, a version of *kpn*2I*.C* expression plasmid containing a 1-bp insertion between the annotated TTG translational start and the putative downstream ATG start codon was created. Cells harboring the pMetGalKpn plasmid and mutated *kpn*2I*.C* expression plasmid formed purple colonies on McConkey agar plates. This result indicates that the mutant *kpn*2I*.C* expression plasmid did not provide functional C.Kpn2I. Therefore, it follows that translation initiation from the ATG codon is not efficient or C.Kpn2I initiated from this site is not functional. We consider this result as an indication that the annotated TTG codon is indeed used to initiate translation of *kpn*2I*.C* mRNA and that therefore C.Kpn2I indeed binds within the coding region of its gene.

### C.Kpn2I strongly bends DNA

**Figure 4.**
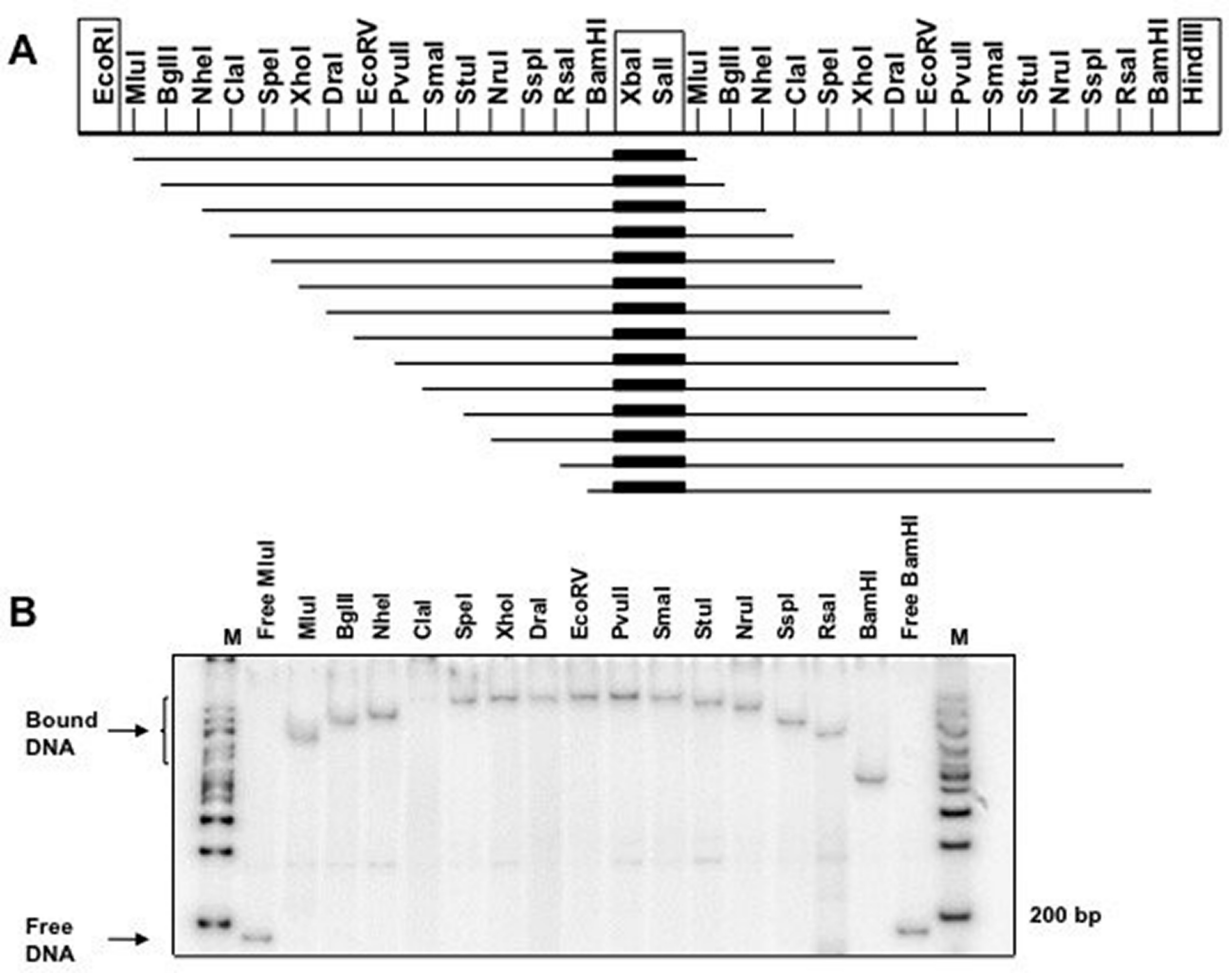
DNA bending by C.Kpn2I. (**A**) The scheme present the 60-bp Kpn2I DNA fragment containing the C.Kpn2I binding site (indicated by a thick black bar) cloned between the XbaI and SalI sites of duplicated multiple cloning site of plasmid pBend2 (40). (**B**) Plasmid aliquots were treated with indicated restriction enzymes, combined with C.Kpn2I and resolved by native PAGE. No C.Kpn2I was added to lanes labeled ‘Free MluI’ and ‘Free BamH’. ‘M’ a molecular weight marker lanes.

The characteristic pattern of protection/hypersensitivity observed in the DNase I footprinting experiments suggested that C.Kpn2I bends DNA (60). To verify this conjecture, a circular permutation test (40) was performed in the presence of high concentrations of C.Kpn2I sufficient to completely convert the DNA into a low-mobility complex. The results are presented in Figure 4. As can be seen, the mobility of C.Kpn2I complexes with DNA containing the binding site strongly depends on the binding site location with respect to DNA fragment ends, which is an indication of a stationary bend introduced by bound C.Kpn2I. Using an equation from (40) we estimate that the bend angle is ∼109°.

### *In vitro* transcription from Kpn2I promoters

*In vitro* transcription with purified σ^70^ RNAP holoenzyme and Kpn2I promoter fragments was performed. No transcription from a DNA fragment containing the *kpn*2I*.R* promoter was detected, confirming that this promoter is very weak.

**Figure 5.**
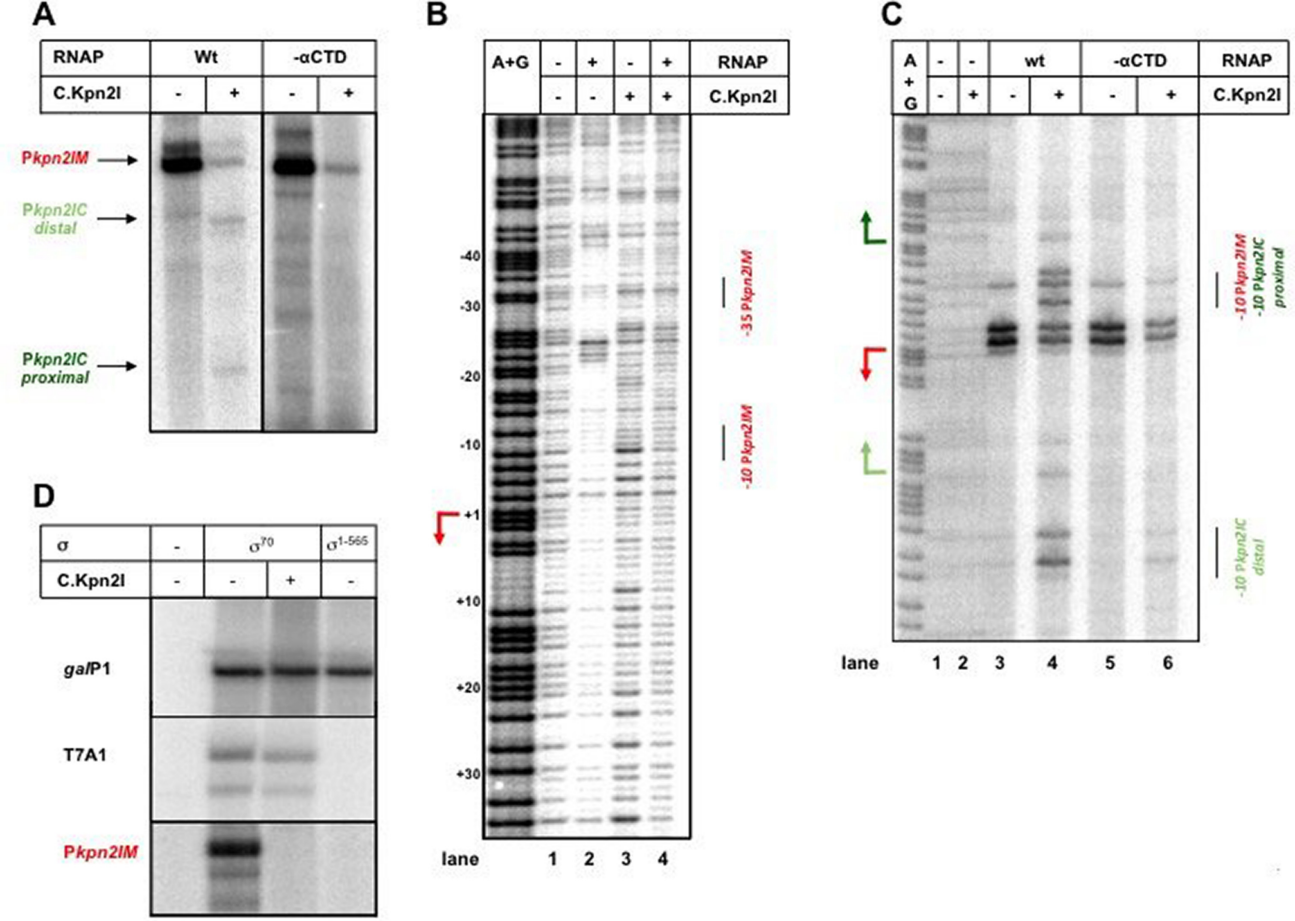
*In vitro* transcription and RNAP promoter complex formation in the presence of C.Kpn2I. (**A**). Results of multiple-round transcription by σ^70^ RNAP holoenzyme with wild-type (‘wt’) α subunit or with α lacking the CTD domain (‘-αCTD’) from a DNA fragment containing the intergenic region between *kpn*2I*.M* and *kpn*2I*.C* in the presence or in the absence of added C.Kpn2I. (**B**) The same DNA fragment was combined with σ^70^ RNAP holoenzyme, C.Kpn2I, or both and subjected to DNase I footprinting. (**C**) As in B but showing the results of KMnO_4_ probing of complexes formed by σ^70^ RNAP holoenzymes with wild-type α or α lacking the CTD. (**D**) Results of multiple-round transcription in the presence or in the absence of C.Kpn2I by RNAP holoenzymes containing wild-type σ^70^ or σ^1–565^ lacking the region 4 domain. In addition to transcription from the *kpn*2I*.M* promoter, results of transcription from strong -10/-35 class promoter T7 A1 and extended –10 class *gal*P1 promoter are shown.

*In vitro* transcription from DNA fragment between *kpn*2I*.M* and *kpn*2I*.C* revealed a single transcript (Figure 5A). Primer extension analysis indicated that this transcript originated from the major *kpn*2I*.M* promoter defined *in vivo*. The addition of recombinant C.Kpn2I strongly decreased the abundance of *kpn*2I*.M* transcripts (Figure 5A) and led to the appearance of two new transcripts, which, based on primer extension analysis, originated from *kpn2I.C* promoters identified *in vivo*.

DNase I footprinting experiment showed that RNAP alone formed a footprint corresponding to promoter complex at the *kpn*2I*.M* promoter (Figure 5B). This complex disappeared upon the addition of the C.Kpn2I.

Upstream of the –35 element of the *kpn*2I*.M* promoter there is an AT-rich sequence that could function as an UP element. This AT-rich segment also serves a C-box, the binding site for C.Kpn2I. Thus, C.Kpn2I may inhibit the *kpn*2I*.M* promoter by acting as an anti-activator and preventing the αCTD interaction with DNA. To test this idea, *in vitro* transcription with mutant σ^70^ holoenzyme lacking αCTD was performed. As can be seen from Figure 5A, the level of *kpn*2I*.M* transcription by this enzyme in the absence of C.Kpn2I was equal to that by the wild-type holoenzyme. Further, transcription by both enzymes was inhibited by C.Kpn2I. KMnO_4_ probing allows one to visualize localized promoter melting at and downstream of the -10 promoter element by monitoring the appearance of permanganate-sensitive single-stranded thymines. In agreement with *in vitro* transcription experiments, both wild-type, and αCTD-less RNAP holoenzymes formed open complexes on *kpn*2I*.M* promoter equally well (Figure 5C). In reactions containing wild-type RNAP, C.Kpn2I decreased open complex formation on *kpn*2I*.M* but stimulated open complex formation on both *kpn*2I*.C* promoters. Open complex formation on *kpn*2I*.M* by αCTD-less RNAP was inhibited by C.Kpn2I but no increase in complex formation on *kpn*2I*.C* promoters was observed. We take these results as evidence that *i*) the *kpn*2I*.M* promoter is not UP element dependent and C.Kpn2I inhibition of *kpn*2I*.M* does not occur by targeting αCTD interactions with upstream DNA, and *ii*) *kpn*2I*.C* promoters require αCTD for full activity.

The C.Kpn2I binding site overlaps with the –35 promoter element of the *kpn*2I*.M* promoter. We considered whether C.Kpn2I may inhibit *kpn*2I*.M* transcription by interfering with σ^70^ region 4 that recognizes the –35 promoter element. To test this, we set transcription reactions with RNAP holoenzyme containing σ^1–565^, a C-terminal deletion variant of σ^70^ lacking region 4 (41). Both wild-type and σ^1–565^ holoenzymes transcribed equally well from the extended –10 class *gal*P1 promoter, as expected (Figure 5D). In contrast, σ^1–565^ holoenzyme did not transcribe from the -10/-35 class T7 A1 promoter. The σ^1-565^ holoenzyme was also inactive on *kpn*2I*.M*. The result is consistent with idea that bound C.Kpn2I interferes with σ^70^ region 4 interactions with –35 element of *kpn*2I*.M* promoter, leading to inhibition of promoter complex formation.

### C.Kpn2I acts as a transcription elongation roadblock

**Figure 6.**
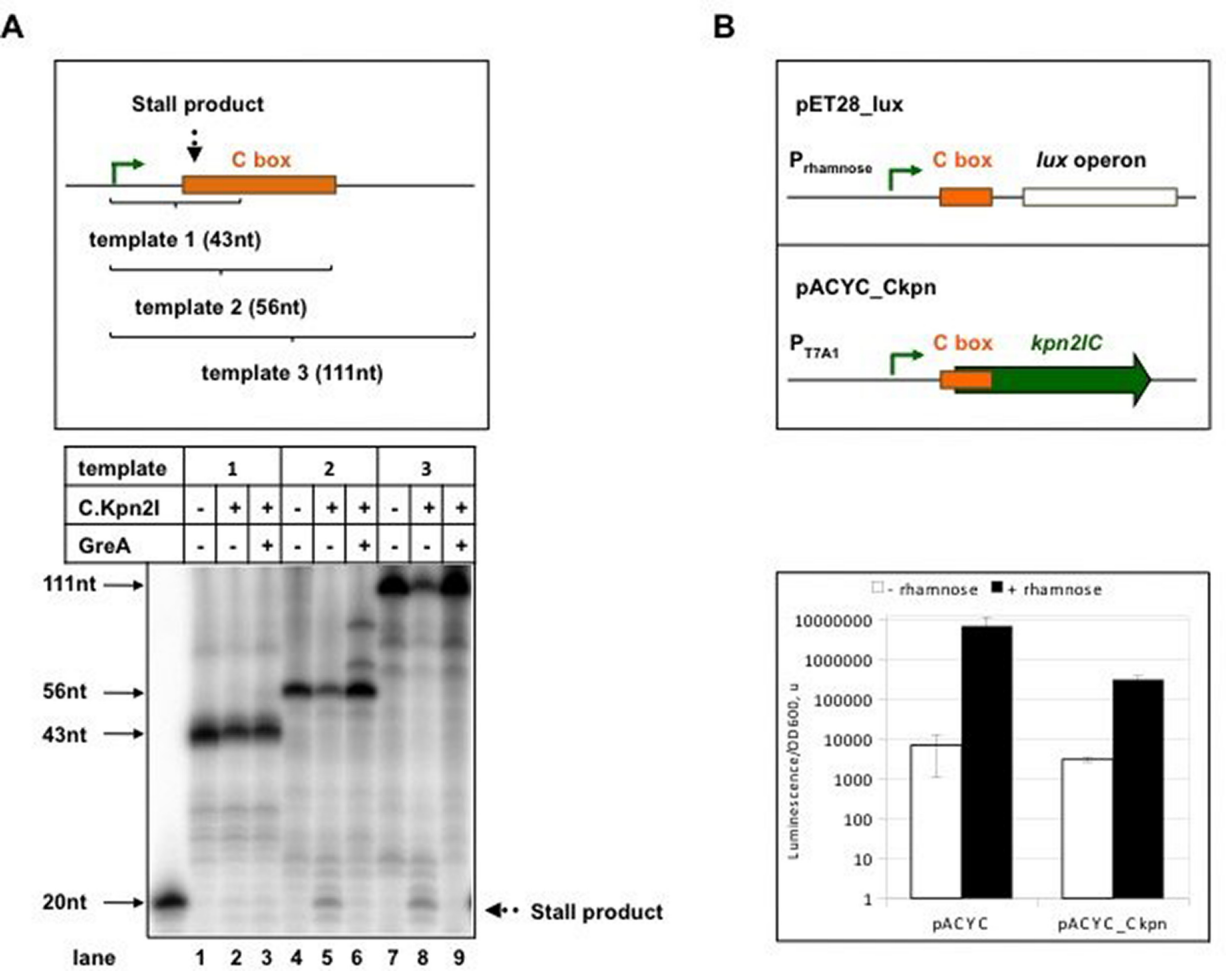
C.Kpn2I binding inhibits transcription elongation through the binding site. (**A**) At the top, three DNA fragments used as templates in *in vitro* transcription reactions are shown. Expected sizes of run-off transcripts and the position of the C.Kpn2I binding site relative to transcription start point (black bent arrow) are indicated. The gel below shows the results of transcription from the three templates in the presence or in the absence of C.Kpn2I. Where indicated, reactions were supplemented with transcript cleavage factor GreA. (**B**) At the top, schemes of plasmids used in *in vivo* experiment are shown. Distances from transcription start site and C.Kpn2I binding site in these constructs are the same as in the Kpn2I system. Levels of luminescence of cells carrying pET28_lux plasmid with a compatible pACYC plasmid with or without the *kpn*2I.*C* gene were measured in the presence or absence of rhamnose. Mean values and standard deviations obtained from four independent experiments are presented.

In all C-protein dependent R–M systems studied to date, production of excess C-protein is prevented by autoinhibition of c gene transcription by C-protein binding to a site that overlaps the promoter (9,20–23,28,31,32,61). The position of the C.Kpn2I binding site, within the annotated *kpn*2I*.C* ORF, is unprecedented, since all C-protein binding sites characterized today are located upstream of C-protein genes promoters (27). Clearly, C.Kpn2I should be unable to control its own production in a way used by other C-proteins. We considered a possibility that binding of C.Kpn2I within its gene may inhibit the elongation of RNA initiated from upstream *kpn*2I*.C* promoters. To test this possibility were fused a strong T7 A1 promoter to DNA transcribed from the *kpn*2I*.C_prox* promoter. The fusion allowed us to exclude the interference from divergent *kpn2I.M* promoter and concentrate on the effects of C.Kpn2I on transcription elongation, which shall be independent on promoter from which transcription is initiated. Three *in vitro* transcription templates were tested. Templates 2 and 3 contain an entire C.Kpn2I binding site and should result in the appearance of run-off transcripts of 56 and 111 nucleotides, correspondingly. Template 1 is truncated at position +43 and does not contain the C.Kpn2I binding site. Multiple-round transcription in the absence of added C.Kpn2I revealed expected transcripts for each of the three templates (Figure 6A). The addition of C.Kpn2I had no effect on transcription from template 1. In contrast, the amount of run-off transcripts from templates 2 and 3 was strongly decreased and a short C.Kpn2I-dependent transcript appeared in reactions containing both templates. Its size was ∼20 nucleotides, consistent with its appearance due to stalling of elongating RNAP by the bound C.Kpn2I.

Stalling on a roadblock causes RNAP backtracking (62). Backtracked complexes can be rescued by the addition of transcript cleavage factors Gre (63). These factors also allow multiple approaches by RNAP to a roadblock, stimulating its bypass (64). Addition of GreA to transcription reactions containing C.Kpn2I led to disappearance of 20-nt blocked transcript and restored the amount of run-off products to levels seen in the absence of C.Kpn2I. We conclude that C.Kpn2I blocks elongating RNAP *in vitro* and propose that *in vivo* C.Kpn2I autoregulates its own synthesis by controlling transcription elongation of its gene.

The *in vivo* demonstration of C.Kpn2I action as a roadblock in the context of intact R–M system is complicated by the facts that i) deregulation of Kpn2I gene expression leads to toxicity (24) and ii) the C.Kpn2I binding site is extensive and is imbedded in the open reading frame that encodes it, making difficult to control the results of mutagenesis. Therefore, to show the capacity of C.Kpn2I to act as a roadblock *in vivo* we fused the initial transcribed sequence of *kpn*2I.*C*, including the entire C-box to luciferase *lux* operon and put the resulting fusion under the control of a rhamnose inducible promoter of pET28_Lux plasmid (Figure 6B). Cells carrying this plasmid were supplemented with a compatible pACYC plasmid with or without the *kpn*2I.*C* gene under control of T7 A1 promoter. In the presence of rhamnose, the level of luminescence of cultures carrying the C.Kpn2I production plasmid was ∼20 lower than in cells carrying pACYC. Thus, C.Kpn2I, whose binding site is located 20 bp downstream of the transcription start point of promoter responsible for luciferase production, has a strong negative effect on luciferase synthesis, consistent with a roadblock mechanism.

### Quantitative modeling of Kpn2I R–M system dynamics

Based on the experimental results, we developed a quantitative model of Kpn2I R–M system. We previously showed that modeling regulation of R–M systems can: (i) explain well *in-vitro* measured transcription activities, for both natural and mutant promoter sequences (31), (ii) reasonably explain *in-vivo* measurements of R–M system dynamics (65), (iii) assess the effects on the system dynamics of perturbing R–M regulatory features (66). The model developed here allows us to visualize and investigate the dynamics that results from regulatory mechanisms operational in the system. Furthermore, the model allows us to *in silico* perturb main regulatory features (e.g. abolish control by C.Kpn2I) and observe the effect of these perturbations on the system dynamics. Such *in-silico* perturbations are particularly important since i) abolishing control by C.Kpn2I experimentally is very hard, as (24) showed that the presence of C.Kpn2I is necessary to eliminate the toxicity of M.Kpn2I, (ii) even if C.Kpn2I control could be abolished experimentally, it would be hard to separate its different regulatory effects (repression of *kpn*2I.*M* transcription initiation from the roadblock effect).

**Figure 7.**
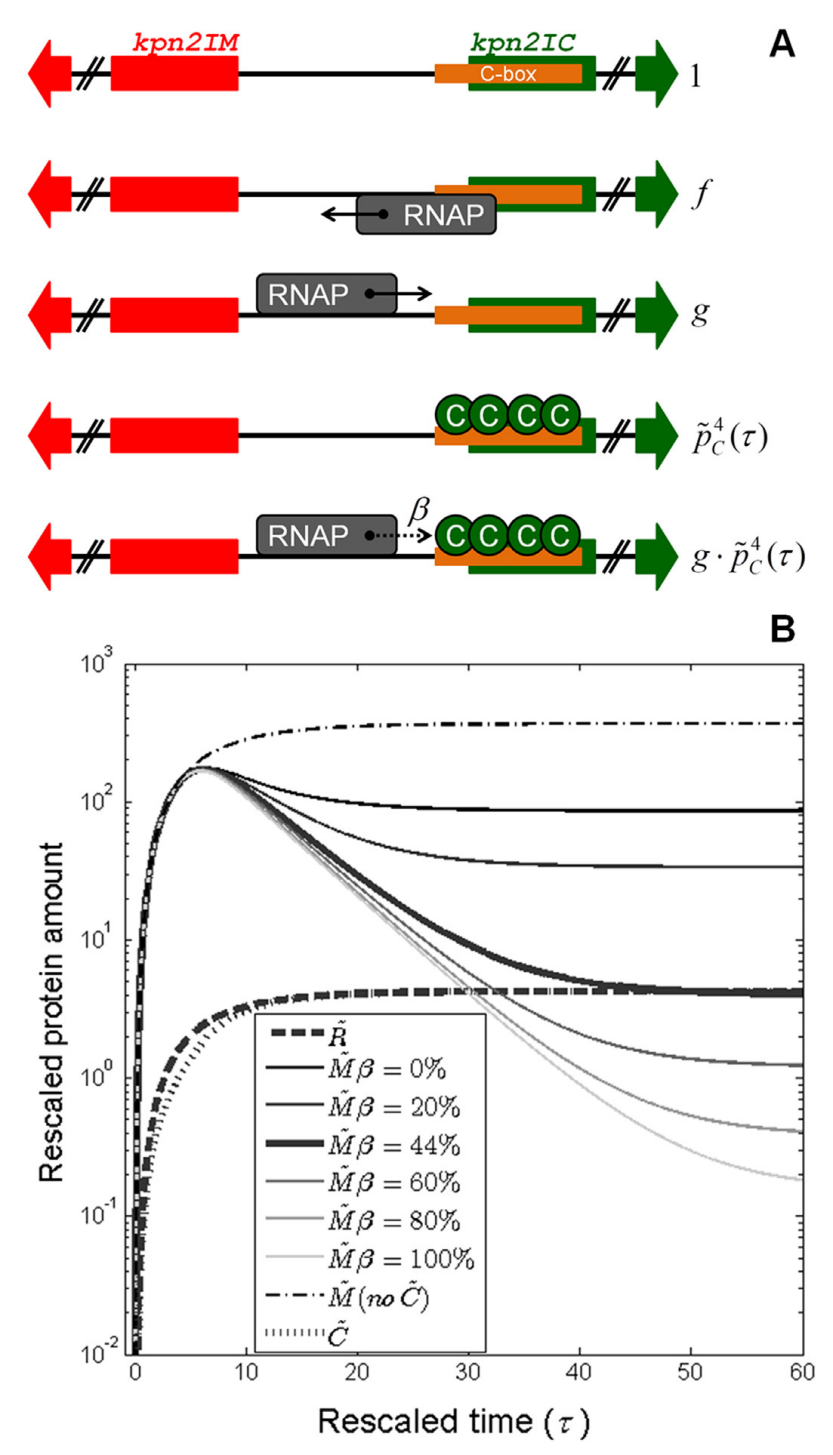
*In silico* prediction of Kpn2I system expression dynamics. (**A**) Modeling Kpn2I system transcription regulation. Allowed configurations of the common regulatory region separating divergently transcribed *kpn*2I.*M* (red arrow) and *kpn*2I.*C* (green arrow) genes are schematically presented, together with their corresponding statistical weights denoted on the right (detailed explanation in the main text). Direction of transcription by RNAP (gray rectangle) bound to a particular promoter is indicated by its associated arrow; the dotted arrow is associated with RNAP transcribing through a roadblock imposed by bound C.Kpn2I proteins (green circles) with probability *β* (indicated in the figure). (**B)** Change of the rescaled protein amounts with time during the system establishment in a naïve host is predicted by the quantitative model; the figure also predicts how appropriate perturbations, i.e., abolishing transcription control by C.Kpn2I, and perturbing the roadblock efficiency (changing *β* value), affect M.Kpn2I dynamics. Thick curves correspond to the wild-type system dynamics for: R.Kpn2I ($\tilde{R},$ thick dashed), C.Kpn2I ($\tilde{C},$ thick dotted), and M.Kpn2I ($\tilde{M}{\rm{ }}\beta = 44\% ,$ thick full). Thin curves correspond to M.Kpn2I dynamics upon the following perturbations: (i) abolishing C.Kpn2I production (dash-dotted curve), (ii) changing *β*, where gradually increasing *β* corresponds to changing the curve shade from the darkest to the lightest – note that $\beta = 44\%$ corresponds to the estimate for the wild-type system.

The rescaled expression dynamics (for scaling, see Methods) of *i*th transcripts $(\tilde{m})$ and proteins $(\tilde{p})$ is described by the following differential equations:

(3.1) \begin{equation*}\begin{array}{@{}l@{}} \frac{{d{{\tilde{m}}_i}(\tau )}}{{d\tau }} = {{\tilde{\varphi }}_i} - {{\tilde{m}}_i}(\tau ){\rm{ }},\\ \frac{{d{{\tilde{p}}_i}(\tau )}}{{d\tau }} = \tilde{k} \cdot {{\tilde{m}}_i}(\tau ) - x \cdot {{\tilde{p}}_i}(\tau ){\rm{ }}, \end{array}\end{equation*}

where $i = R,M,C$ (appearing in the subscript of the quantities in Equation (3.1)) stands for restriction endonuclease, methyltransferase and C-protein, respectively; ${\tilde{\varphi }_i},$$\tilde{k},$$x$ and $\tau$ are, respectively, rescaled transcription activity, rate of translation, rate of protein degradation, and time (see Methods). The first terms on the right-hand side of the equations quantify synthesis of transcripts/proteins, while the second terms describe their decay. We have chosen to work with rescaled quantities, as this reduces the number of parameters in the model and we are not concerned with absolute transcript and protein amounts at the current approximation (note however that the relative transcript/protein amounts can be directly compared with each other in Figure 7B). Also, $\tilde{k}$ and $x$ are assumed equal for *R, M* and *C*, so the differences in protein dynamics are a direct reflection of differences in transcription level.

We next thermodynamically modeled Kpn2I transcription regulation (see Materials and Methods), based on the classical Shea-Ackers assumption that promoter transcription activity $({\tilde{\varphi }_i})$ is proportional to its equilibrium occupancy by RNAP (67). In particular, if we look at the common regulatory region separating divergently transcribed *kpn*2I*.M* and *kpn*2I*.C* genes, the following five configurations (characterized by the statistical weights indicated below) are possible (see Figure 7A):

- i. $1$ – empty DNA;
- ii. $f$ – RNAP bound to the *kpn*2I*.M* promoter;
- iii. $g$ – RNAP bound to *kpn*2I*.C* promoter(s) (note that RNAP cannot be bound to *kpn*2I*.M* and *kpn*2I*.C* promoters at the same time due to their partial overlap);
- iv. $\tilde{p}_C^4(\tau )$ – C.Kpn2I tetramer bound to a site partially overlapping the *kpn*2I*.C* gene excluding RNAP binding to *kpn*2I*.M* promoter (we here take into account only C.Kpn2I binding in the form of tetramer, since the regulatory role of C.Kpn2I dimer interaction with half-sites is unknown);
- v. $g \cdot \tilde{p}_C^4(\tau )$ – RNAP bound to *kpn*2I*.C* promoter(s) in the presence of bound C.Kpn2I tetramer.

From these configurations (statistical weights), and the Shea-Ackers assumption, we obtain the following expression for *kpn*2I*.M* promoter transcription activity:

(3.2) \begin{equation*}{\tilde{\varphi }_M}({\tilde{p}_C}) = \frac{{\tilde{\alpha } \cdot f}}{{1 + f + g + \left( {1 + g} \right) \cdot \tilde{p}_C^4(\tau )}}{\rm{ }}.\end{equation*}

To infer the transcription activity of *kpn*2I*.C* promoter, note that the roadblock effect due to C.Kpn2I tetramer binding effectively decreases the rate of transcription from *kpn*2I*.C* promoter(s). The effective *kpn*2I*.C* transcription is thus proportional to the probability of initiating a transcript (which is determined by the appropriate statistical weights, through the Shea-Ackers approach), multiplied by the probability of having a complete transcript elongation, which requires that RNAP reads through the C.Kpn2I roadblock (see Methods for more details on the expression below):

(3.3) \begin{equation*}{\tilde{\varphi }_C}({\tilde{p}_C}) = \frac{{\tilde{\alpha } \cdot g \cdot \left( {1 + \tilde{p}_C^4(\tau )} \right) \cdot \left( {1 + f + g + \beta \cdot \left( {1 + g} \right) \cdot \tilde{p}_C^4(\tau )} \right)}}{{{{\left( {1 + f + g + \left( {1 + g} \right) \cdot \tilde{p}_C^4(\tau )} \right)}^2}}}{\rm{ }},\end{equation*}

where the parameter $\beta$ corresponds to the fraction of elongating RNAP that reads through. The rescaled rate at which RNAP leaves a promoter once bound $(\tilde{\alpha })$ is assumed to have the same value for *kpn*2I*.M* and *kpn*2I*.C* promoters (consistent with the Shea-Ackers assumption). As transcription of *kpn*2I*.R* is constitutive, ${\tilde{\varphi }_R}$ is a constant. The model parameters are estimated from experimental data, as described in Materials and Methods.

The protein expression dynamics, which is predicted for intact Kpn2I system by our model, is presented in Figure 7B (thick curves). As one can see, the model, together with parameters inferred from experimental data, provides for a much lower early expression of R.Kpn2I with respect to M.Kpn2I. In the early phase of the system establishment, the amount of M.Kpn2I rapidly reaches a peak level, and then decays to a lower steady-state level. Similar massive production of methyltransferase at early times after R–M system entry in naïve cells was observed during *in vivo* measurements of Esp1396I R–M system dynamics (65).

To understand the role of the regulation by C.Kpn2I, we investigated the effect of abolishing C.Kpn2I production (so that the regulation of the system by C.Kpn2I is completely absent) on M.Kpn2I dynamics. This corresponds to setting $\tilde{p}_C^{}(\tau ) = 0$ in Equations (3.2) and (3.3). In this case, transcription from *kpn*2I*.M* promoter is limited only by the competition due to RNAP binding to the overlapping *kpn*2I*.C* promoter, which results in the thin dash-dotted curve shown in Figure 7B.

Next, to understand the significance of the roadblock mechanism, we varied $\beta$ in the model (Equation (3.3)) from 0% (no RNAP elongates through bound C.Kpn2I) to 100% (no roadblock). As can be seen from Figure 7B, for $\beta = 0\%$ the M.Kpn2I dynamics comes close to the curve obtained when C.Kpn2I does not regulate the system at all (see the dash-dotted curve in the Figure 7B). This is a consequence of the fact that when RNAP cannot elongate through bound C.Kpn2I, only a small amount of C.Kpn2I is generated, which only weakly represses transcription of *kpn*2I*.M*.

## DISCUSSION

In this work, we characterize transcription regulation in C-protein dependent R–M system Kpn2I. DNA-binding C-proteins regulate gene expression in numerous Type II R–M systems. During establishment of R–M system genes in a naïve host, C-proteins bind to single or duplicated DNA sites located upstream or partially overlapping with R–M system genes promoters and orchestrate a cooperative time-delayed switch from *met* to *res* gene transcription, ensuring that the host DNA is not attacked by prematurely synthesized restriction endonuclease. After an R–M system has established itself in host bacterium, C-proteins ensure that no excess methyltransferase that could compromise defense from foreign DNA is synthesized. C-proteins also regulate transcription of their own gene homeostatically maintaining a steady-state concentration that in turn determines the optimal relative amounts of restriction endonuclease and methyltransferase transcripts production. Overall, the logic of gene expression control by known C-proteins resembles that of phage λ repressor, a paradigmal DNA binding transcription initiation factor that orchestrates a switch between lytic and lysogenic development of a virus by modulating intrinsic RNAP affinity to different promoters (68).

The most unexpected finding of our work is that C.Kpn2I protein controls its own synthesis and, as a result, ensures optimal accumulation of M.Kpn2I in an entirely different way. We show that the C.Kpn2I binding site is located inside the *kpn*2I.*C* gene open reading frame. While the location of the binding site allows for ‘standard’ regulation of *kpn*2I*.M* promoter transcription, it makes impossible autoregulation of *kpn*2I.*C* transcription at the initiation stage. Instead, bound C.Kpn2I acts as a roadblock to RNAP transcribing its own gene, thus decreasing production of *kpn*2I.*C* mRNA. Elongating RNAP is a powerful molecular motor (69). A sharp stationary bend introduced by C.Kpn2I may help to stall the transcription complex. While *in vivo* efficiency of the roadblock (*β*) is presently impossible to estimate, *in vitro*, at standard transcription conditions, almost half of transcribing RNAPs are blocked by bound C.Kpn2I, i.e. *β* estimated from the experimental data by using the model is ∼0.44.

While the mechanism of autoregulation by C.Kpn2I is highly unusual, the functional consequences appear to be very much in line with those of more conventional C-proteins. As can be seen from Figure 7B, in the absence of C.Kpn2I, M.Kpn2I reaches significantly higher amounts at steady-state. It was previously shown (24), that toxicity of M.Kpn2I causes low transformation efficiency in the absence of C.Kpn2I. Modeling results in Figure 7B are consistent with C.Kpn2I absence being associated with elevated steady-state levels of M.Kpn2I.

Thus, as is also the case in other systems, C.Kpn2I limits the steady-state amount of M.Kpn2I, while allowing massive M.Kpn2I accumulation at early times, necessary for the host genome protection from gradually accumulating R.Kpn2I. Furthermore, the requirement for the amount of C.Kpn2I to be high enough to efficiently repress *Kpn*2I*.M* transcription imposes a constraint on the efficiency of the roadblock effect. Specifically, while the roadblock efficiency is high (see above), it is still far from being complete, which allows establishing the usual pattern of R–M systems expression dynamics – i.e., note that *β* = 0% would lead to a much higher M.Kpn2I steady-state level, and the near-absence of the characteristic peak in M.Kpn2I accumulation at early time (see Figure 7B). Our data indicate that transcript cleavage Gre factors may modulate the *β* parameter. In Figure 7B we see that such modulation (i.e. varying the *β* value), is predicted to significantly change the steady-state amount of M.Kpn2I, so Gre factors may in this way modulate the protective function of Kpn2I.

One should note that in addition to the repressing effect due to the roadblock, C.Kpn2I also indirectly activates its own synthesis. That is, binding of C.Kpn2I to DNA inhibits RNAP binding to major *kpn*2I*.M* promoter, indirectly activating transcription from overlapping *kpn*2I*.C* promoters. These two opposing (i.e. activating and repressing) effects of C.Kpn2I on its own synthesis evidently serve to adjust the steady-state levels of the control protein. Such combination of the activating and the repressing effects is also exhibited in other R–M systems (see e.g. (59)) where binding of C protein dimer on the promoter distal position first activates transcription, while the subsequent binding to the promoter proximal position leads to its repression. For C.Kpn2I, this activation and repression are accomplished, respectively, by the overlapping promoter and the roadblock effects discussed above.

Overall, two mechanistically different modes of regulation, one exhibited in Kpn2I, and other exemplified by other C-protein dependent systems, lead to essentially the same regulation of protein dynamics, necessitated by functional requirements during R–M system establishment in naïve host and subsequent maintenance. Since R–M systems rely on precise temporal regulation of toxic gene expression, the results presented here contribute to our understanding of mechanisms through which such regulation can be achieved and highlight the versatility of C-proteins in affecting different stages of the transcription cycle, making them attractive tools for synthetic biology applications.

**Figure 8.**
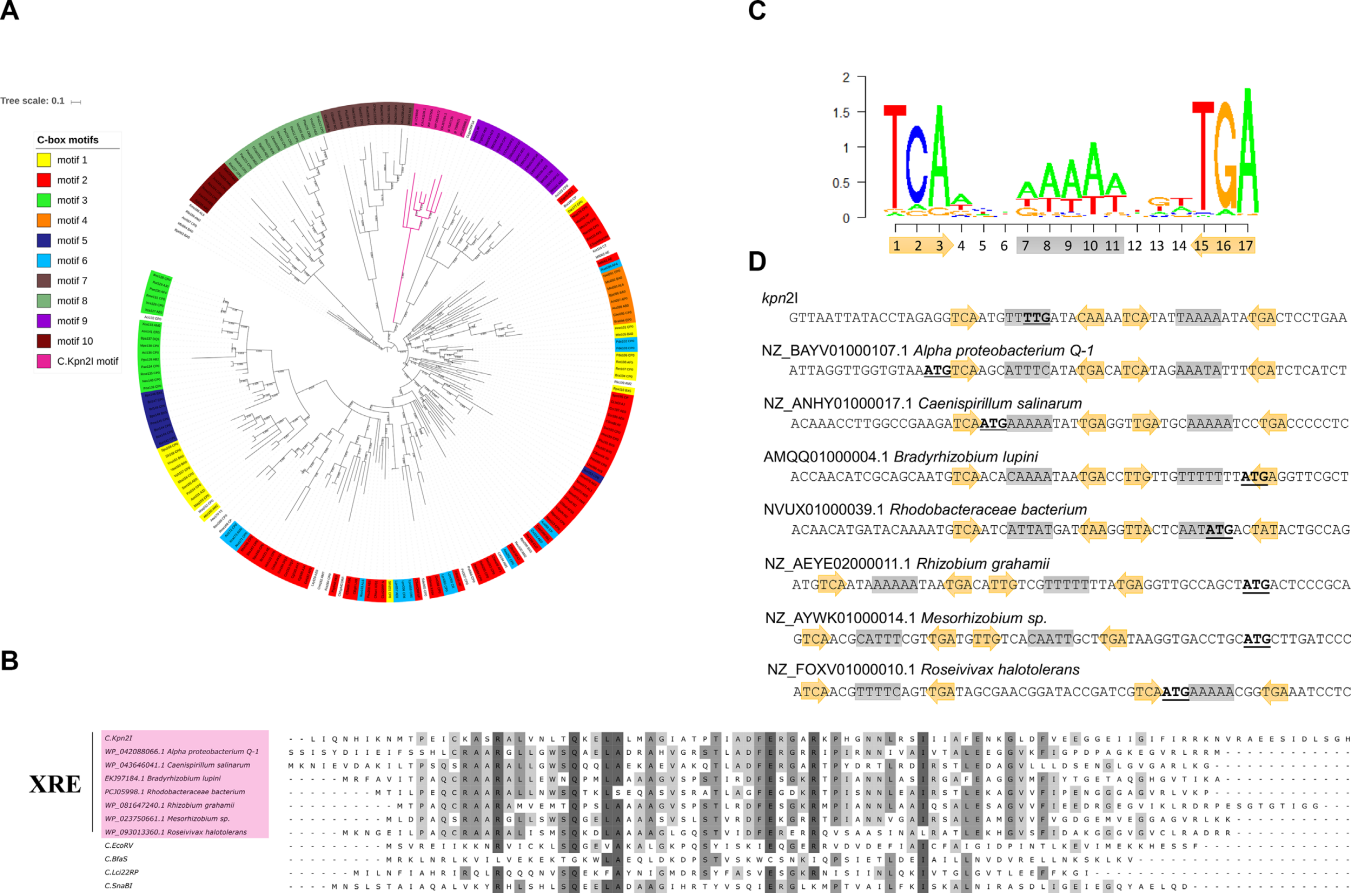
Phylogenetic analysis of C.Kpn2I and prediction of C-box for C.Kpn2I-like proteins. (**A**) Maximum likelihood tree built from C-protein sequences collection from (27) and C.Kpn2I, and several C.Kpn2I-like proteins. Colors indicate C proteins whose predicted binding sites fall into distinct motifs (1 through 10, as defined by Sorokin *et al.* The C.Kpn2I-like proteins clade is colored magenta. (**B**) Multiple alignment of C.Kpn2I, C.Kpn2I-like XRE proteins, and several C-proteins. Conserved residues are indicated by various shades of gray, with darker hue showing higher extent of conservation. (**C**) A C.Kpn2I-motif logo. Below, the positions occupied by the outside inverted trinucleotide repeat are shown as ochre arrows, the position of the central A/T rich track is shown on grey background. (**D**) The locations of C.Kpn2I-motifs with respect to the beginning of C.Kpn2I coding sequence and the sequences of XRE proteins shown in B are presented. The annotated translation start sites are highlighted in bold typeface and are underlined. The elements of the C.Kpn2I-motif are shown as in C.

Given how widespread are C-protein controlled systems (27), a question arises whether the C.Kpn2I mechanism of action is unique and how is this protein related to other C-proteins. Earlier, we performed a comprehensive phylogenetic analysis of C-proteins and predicted 10 distinct binding sites (27). However, the binding sites were predicted by inspecting non-coding regions upstream of C-protein coding genes, which by default excluded C.Kpn2I and other proteins that may act similarly. Results of extended analysis of currently available C-protein sequences from public databases is presented in Figure 8A. First, we found that in addition to the Kpn2I system originally discovered in *K. pneumoniae*, virtually identical R–M systems exist in *Cronobacter sakazakii* (GI 1126564860) and *E. coli* 7748_7#48 (GI 487672847). The sequences of their C-proteins differ in one and three aminoacid positions, respectively, from the C.Kpn2I sequence. The predicted binding sites of C-proteins are located inside the C-proteins genes and differ from Kpn2I C-box in just one position. Second, we observed that there are several uncharacterized R–M systems with high degree of sequence identity to R.Kpn2I and M.Kpn2I (68–81%), which do not contain any recognizable C-protein gene. Thirdly, among the R–M systems that are clearly related to Kpn2I (40–65% aminoacid sequence identity to R.Kpn2I and/or M.Kpn2I), there are systems that contain adjacent genes coding for XRE family transcription factors (49). These systems, unlike Kpn2I, are encoded in bacterial genomes and many are located close to tRNA or integrase genes (Supplementary Figure S3). The latter observation makes it likely that they have arisen in their current locations due to horizontal gene transfer, which may have been responsible for observed variety of genetic organization.

C.Kpn2I and closely related sequences form a distinct branch on the C-protein phylogenetic tree (Figure 8A). In fact, C.Kpn2I is very distantly related to most known C-proteins (maximal aminoacid identity 29%) and is a closer relative of XRE proteins (maximal aminoacid identity 41%), many of which are not associated with R–M systems (Figure 8B). Analysis of corresponding DNA sequences, both upstream and downstream of the annotated translation start sites, identified a distinct motif with an outside three-nucleotide inverted repeat and a central A/T rich segment (Figure 8C). Sequences coding for C.Kpn2I-like proteins contain either a single, or a duplicated (as is the case of C.Kpn2I) motif (Figure 8D). Interestingly, the motif is either entirely or partially located in the beginning of annotated open reading frames or immediately upstream (Figure 8D). These findings suggest that the mode of transcription autoregulation via elongation roadblock is common for C.Kpn2I-like proteins. One can make an argument that a transcription regulator that controls its own synthesis through the binding to its own gene provides the most economical, promoter-independent autoregulatory system that is perfectly suited for horizontal gene transfer.

## Supplementary Material

Supplementary Data
