# Supplementary material for: Controller protein of restriction–modification system Kpn2I affects transcription of its gene by acting as a transcription elongation roadblock
Source: Nucleic Acids Res. 2018 Oct 8;46(20):10810–26. doi: 10.1093/nar/gky880 (PMC6237814; doi:10.1093/nar/gky880)
Supplement: Supplementary Data [file gky880_supplemental_files.docx]

**SUPPLEMENTARY DATA**

**Supplementary Figure S1.**

**
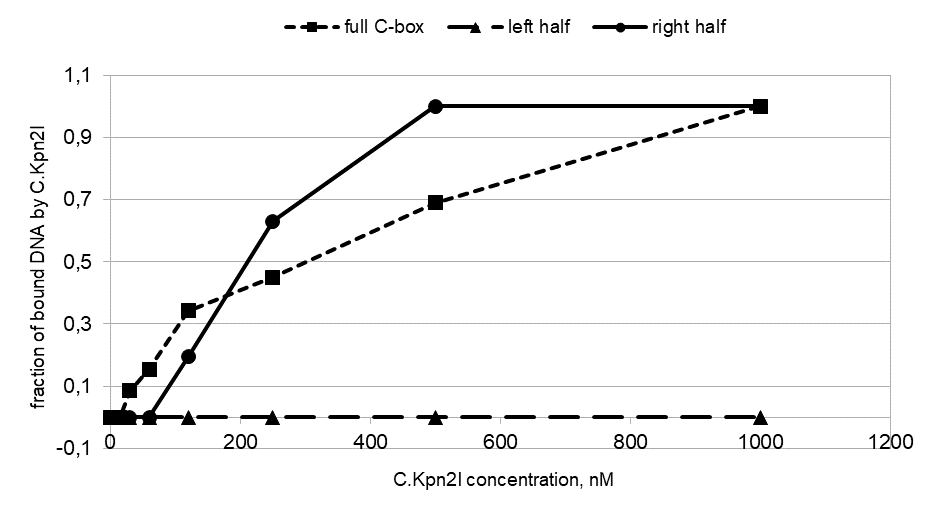
**

Quantification results of EMSA gels (Fig. 3D) with double-stranded Kpn2I DNA fragment that contains full C-box (“full C-box”) or shorter fragments corresponding to its left- and right-hand side halves (“left half” and “right half”).

**Supplementary Figure S2.**

**
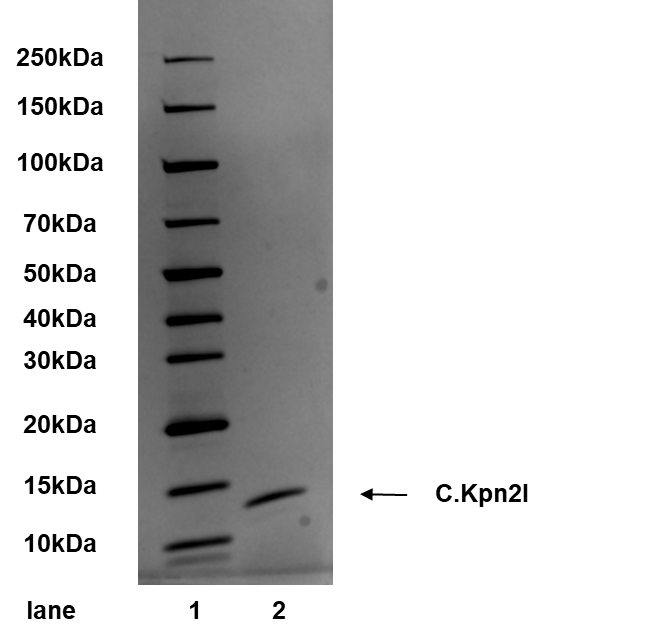
**

A Coomassie stained SDS gel showing purity of C.Kpn2I used throughout this work. Lane 1 – PageRuler Broad Range Unstained Protein Ladder (Thermo Scientific), lane 2 – C.Kpn2I sample after purification.

**Supplementary Figure S3.**

**Genetic organization of putative R-M systems encoding enzymes similar to Kpn2I R-M system enzymes.** The genetic architecture of the Kpn2I R-M system is schematically shown at the top (see also Figure 1). Below, loci containing similarly arranged R-M genes with source bacteria listed and the extent of aminoacid sequence identity to Kpn2I enzymes indicated is shown. Arrows flanking the central R-M gene arrangement show neighboring annotated genes with their putative functions indicated.
